# Holliday junction resolution by *At*-HIGLE: an SLX1 lineage endonuclease from *Arabidopsis thaliana* with a novel in-built regulatory mechanism

**DOI:** 10.1093/nar/gkac239

**Published:** 2022-04-12

**Authors:** Prabha Verma, Poonam Kumari, Shreya Negi, Gitanjali Yadav, Vineet Gaur

**Affiliations:** National Institute of Plant Genome Research, Aruna Asaf Ali Marg, New Delhi 110067, India; National Institute of Plant Genome Research, Aruna Asaf Ali Marg, New Delhi 110067, India; National Institute of Plant Genome Research, Aruna Asaf Ali Marg, New Delhi 110067, India; National Institute of Plant Genome Research, Aruna Asaf Ali Marg, New Delhi 110067, India; National Institute of Plant Genome Research, Aruna Asaf Ali Marg, New Delhi 110067, India

## Abstract

Holliday junction is the key homologous recombination intermediate, resolved by structure-selective endonucleases (SSEs). SLX1 is the most promiscuous SSE of the GIY-YIG nuclease superfamily. In fungi and animals, SLX1 nuclease activity relies on a non-enzymatic partner, SLX4, but no SLX1-SLX4 like complex has ever been characterized in plants. Plants exhibit specialized DNA repair and recombination machinery. Based on sequence similarity with the GIY-YIG nuclease domain of SLX1 proteins from fungi and animals, *At*-HIGLE was identified to be a possible SLX1 like nuclease from plants. Here, we elucidated the crystal structure of the *At*-HIGLE nuclease domain from *Arabidopsis thaliana*, establishing it as a member of the SLX1-lineage of the GIY-YIG superfamily with structural changes in DNA interacting regions. We show that *At*-HIGLE can process branched-DNA molecules without an SLX4 like protein. Unlike fungal SLX1, *At*-HIGLE exists as a catalytically active homodimer capable of generating two coordinated nicks during HJ resolution. Truncating the extended C-terminal region of *At*-HIGLE increases its catalytic activity, changes the nicking pattern, and monomerizes *At*-HIGLE. Overall, we elucidated the first structure of a plant SLX1-lineage protein, showed its HJ resolving activity independent of any regulatory protein, and identified an in-built novel regulatory mechanism engaging its C-terminal region.

## INTRODUCTION

Homologous recombination is one of the fundamental cellular processes in generating genetic diversity and repairing damaged DNA (1). One key intermediate during homologous recombination is a four-way joint DNA molecule: Holliday junction (HJ) (2–4). HJ is one of the many types of joint (branched) DNA molecules appearing during DNA metabolism (Supplementary Figure S1). An inability to process HJs results in genomic instability (5,6). HJs are processed either by dissolution or resolution (7,8). Dissolution involves the formation of a hemicatenated intermediate followed by a dissolvasome catalyzed decatenation resulting in non-crossovers. A dissolvasome comprises helicase, topoisomerase, and structural proteins (9). In contrast, HJ resolution involves structure-selective endonucleases (SSEs) that hydrolyze phosphodiester bonds near the crossover points of HJ, resulting in crossover and non-crossover products. While dissolution is a safeguarding mechanism to circumvent the loss of heterozygosity (3,10,11), resolution plays an essential role in generating genetic diversity (12).

SSEs are present in all spheres of life, exhibiting structural and functional diversity (13). SSEs participating in HJ resolution are known as resolvases (canonical and non-canonical) (14–16). Canonical resolvases make a pair of coordinated symmetrical ligatable nicks across the HJ (e.g. RuvC, Yen1, GEN1 and SEND1). Non-canonical resolvases introduce non-ligatable asymmetrical indentations, requiring further processing before ligation (e.g. MUS81-EME1, SLX1) (12). The structure and catalytic mechanisms of various resolvases have been extensively studied in bacteria, fungi and humans. RuvC is the best characterized canonical resolvase offering a mechanistic paradigm for eukaryotic HJ resolution (17,18). RuvC exists as a homodimer and introduces two symmetrical nicks within the lifetime of a single protein-DNA complex (19–21). GEN1 in humans (Yen 1 in *Saccharomyces cerevisiae*), a member of the Rad2/XPG family (22), exists as a monomer in solution and dimerizes on binding an HJ to facilitate two symmetrical nicks (23). MUS81-EME1 and SLX1-SLX4 in humans are two non-canonical resolvases working together while resolving an HJ (24–34). SLX1 generates the first nick followed by a counter nick by MUS81-EME1 (26,35,36).

SLX1 belongs to the GIY-YIG nuclease superfamily. Besides SLX1, the GIY-YIG superfamily comprises many restriction endonucleases, homing nucleases, and bacterial NER (**N**ucleotide **E**xcision **R**epair) protein UvrC (37). Unlike other resolvases, SLX1 from animals and fungi is proficient in processing a vast repertoire of joint DNA molecules (e.g. HJ, Replication forks [RF], 5′ flaps, 3′ flaps, splayed arm DNA [SA],*etc*.) (Supplementary Figure S1). No other members of the GIY-YIG nuclease family possess such a broad spectrum of substrate specificity. In all reports available to date, SLX1 has to interact with a non-enzymatic protein for its activation (38–40). SLX4 has been reported not only to coordinate the activities of SLX1 and MUS81-EME1 during HJ resolution (26,35,36) but also to serve as an interacting scaffold for XPF-ERCC1, mismatch repair proteins, and proteins involved in telomere maintenance (25–28). Mutations in SLX4 result in a subtype of Fanconi anaemia in humans (41,42).

Compared to animal and fungal resolvases, the biochemical and structural information on plant resolvases is only fragmentary (43). Although the DNA repair and recombination mechanisms are conserved among animals and plants, there are significant differences that may be unique to the plant kingdom, but have not been fully explored. Several plant resolvases have been identified based on sequence similarities with resolvases from fungi and animals. For example, plants have two homologs of GEN1: GEN1 and SEND1 (44–46). Both GEN1 and SEND1 resolve HJs by two symmetrical nicks. The MUS81–EME1 complex from *Arabidopsis thaliana* (*At*-MUS81-EME1), similar to human MUS81-EME1, prefers nicked HJ (47–50), imposing a need of having another resolvase that can generate the initial nick, a function carried out by the SLX1-SLX4 complex in humans. Interestingly, neither SLX1 nor SLX4 like protein has been reported from plants. A nuclease belonging to the GIY-YIG superfamily has been identified from *A. thaliana* (*At-*HIGLE: **H**YL-1 **I**nteracting **G**IY-**Y**IG **L**ike **E**ndonuclease) (AT2G30350) (51). A BLAST (**B**asic **L**ocal **A**lignment **S**earch **T**ool) search (52) identifies *At*-HIGLE as an excinuclease ABC (C subunit), providing clues regarding the possible function of *At*-HIGLE similar to fungal and mammalian SLX1. We began work on the premise that *At*-HIGLE may be a good candidate for plant SLX1.

In the present work, we demonstrate the HJ resolution potential of *At*-HIGLE, which is analogous to SLX1. We also describe a crystal structure of the nuclease domain of *At*-HIGLE, which in turn allowed us to reaffirm *At*-HIGLE as a member of the SLX1 subfamily of GIY-YIG endonucleases. Our data provide insights into the *At*-HIGLE substrate specificity, mode of substrate binding, as well as catalysis, advancing research in plant DNA repair and recombination. Furthermore, we find a novel regulatory mechanism involving the extended C-terminal region of *At*-HIGLE in delimiting its catalytic activity.

## MATERIALS AND METHODS

### Cloning, protein expression and purification

The gene coding for *At*-HIGLE from *Arabidopsis thaliana* (1119 bp) (*At2g30350*) was codon-optimized (BIOMATIK, Canada) for expression in *E. coli*. The vector was sub-cloned into pET28a vector (Novagen) with N-terminal 6x His followed by a SUMO Protease cleavage site and transformed into BL21 (DE3) RIL (Novagen). *At*-HIGLE^1–183^ and *At*-HIGLE^184-368^ and point substitutions were introduced by Quick change Site-directed mutagenesis (Agilent) (Supplementary Table S1). For protein expression, cells were grown in LB medium at 37°C, induced with 0.4 mM isopropyl beta-d-1-thiogalactopyranoside (IPTG) at OD_600_ = 0.6–0.8, and grown overnight at 16°C. Cells were harvested by centrifugation at 5000 rpm for 20 min at 4°C.

For protein purification, bacterial pellet from 2 l culture was resuspended in lysis buffer containing 20 mM Tris–HCl (pH 8.5), 10% glycerol, 5 mM β-mercaptoethanol, 5 mM Imidazole, 0.5 M NaCl and 25 mM L-arginine supplemented with PMSF, lysozyme and protease inhibitor cocktail (SIGMA). The suspension was incubated for 30 min on ice, followed by sonication. The supernatant was collected by centrifugation at 17,000 rpm for 40 min. Soluble fraction was loaded onto His-Trap HP 5 ml column (GE Healthcare) equilibrated with (20 mM Tris–HCl (pH 8.5), 10% glycerol, 5 mM β-mercaptoethanol, 5 mM Imidazole, 0.5 M NaCl and 25 mM l-arginine). Protein was eluted on Akta-FPLC with a 100–1000 mM Imidazole gradient. Peak fractions were analyzed on 12% sodium dodecyl sulfate (SDS)-PAGE. Fractions containing protein of interest were pooled together and digested overnight with SUMO Protease to remove His-tag. Digestion was confirmed by running 12% SDS-PAGE. The digested protein was concentrated using 10 kDa centricon (Amicon, Merck) and injected onto a Sephacryl 16/60 S-200 (GE Healthcare) column equilibrated with (20 mM Tris (pH 8.5), 5% glycerol, 5 mM β-mercaptoethanol and 0.5 M NaCl). The respective peak was confirmed through 12% SDS-PAGE, concentrated, and stored at −20°C in 50% glycerol. Only proteins with *A*_260_/*A*_280_ ratio less than 0.7 were used in the further experiments. All the mutants of *At*-HIGLE were expressed and purified using a similar procedure. For the purification of *At*-HIGLE^1–183^ and *At*-HIGLE^184–368^, SUMO digestion was followed by a second nickel purification before gel-filtration chromatography on Sephacryl 16/60 S-100 (GE Healthcare). *At*-HIGLE^184–368^ culture was lysed in 20 mM Tris–HCl (pH 7.0), 10% glycerol, 5 mM β-mercaptoethanol, 5 mM Imidazole, 0.05% Tween 20, 25 mM l-arginine and 0.5 M NaCl.

### Crystallization and structure elucidation

*At*-HIGLE^1–183^ crystals were obtained by sitting drop vapor diffusion method at a final concentration of 9 mg ml^–1^ at 20°C in 4.0 M Sodium formate. Crystals were cryoprotected with 30% glycerol (v/v) before data collection. X-ray diffraction data were collected at beamline ID-23-2 at European Synchrotron Radiation Facility (ESRF) at a wavelength of 0.8731Å. The crystals belonged to *P*2_1_2_1_2_1_ space group and diffracted to a maximum resolution of 1.7 Å. Diffraction data was processed and scaled using XDS (53). Phases were determined using molecular replacement in the Phase-MR module of Phenix (54). *Cg*-SLX1 was used as a starting model for molecular replacement (PDB: 4XM5) (40). There is one molecule per asymmetric unit. The model was refined iteratively using COOT and PHENIX to an *R*_work_/*R*_free_ of 16.37% and 19.38% at 1.7 Å with 10% reflections for *R*_free_ calculation (55,56). In the final model, 98.0% amino acid residues resided in the allowed region of the Ramachandran plot. The structure validation was carried out using MolProbity (57). Structure analysis was performed using PyMOL (The PyMOL Molecular Graphics System, Version 2.0 Schrödinger, LLC.). Structure-based sequence alignments were done using UCSF Chimera (58). All other sequence alignments were done using CLUSTAL W (59). The diffraction and refinement statistics are summarized in Table 1. The structure was deposited in PDB with accession code 7WME.

**Table 1.** Diffraction and refinement statistics

Diffraction data	*At*-HIGLE^1–183^
Wavelength (Å)	0.8731Å
Resolution range (Å)	32.29–1.7 (1.761–1.7)
Space group	*P* 21 21 21
Unit cell (Å) (*a*, *b*, *c*)	40.52, 53.45, 69.95
Total reflections	217 610 (22 371)
Unique reflections	17 294 (1712)
Multiplicity	12.6 (13.1)
Completeness (%)	99.94 (100.00)
Mean *I*/sigma (*I*)	15.86 (3.45)
Wilson *B*-factor	16.69
*R*-merge	0.1223 (0.6415)
Refinement and structure model	
Reflections used in refinement	17 292 (1712)
Reflections used for *R*-free	1730 (171)
*R*-work (%)	16.37 (18.36)
*R*-free (%)	19.38 (23.11)
Number of non-hydrogen atoms	1354
Macromolecules	1222
Ligands	1
Solvent	131
Protein residues	153
RMS (bonds)	0.014
RMS (angles)	1.31
Ramachandran favored (%)	98.01
Ramachandran allowed (%)	1.99
Ramachandran outliers (%)	0.00
Rotamer outliers (%)	0.80
Clashscore	2.07
Average *B*-factor (Å^2^)	18.57
Macromolecules (Å^2^)	17.38
Ligands (Å^2^)	37.86
Solvent (Å^2^)	29.59
PDB code	7WME

Statistics for the highest-resolution shell are shown in parentheses.

### Nuclease assays

The synthetic DNA substrates for nuclease assays were prepared by annealing DNA oligonucleotides synthesized by Eurofins, India. The oligonucleotides sequences and various oligonucleotides used for annealing substrates are presented in Supplementary Table S2. The substrates comprised of 100 nM unlabeled substrate and 25 nM labeled substrate. Labeled substrate comprised of 6-FAM on X0-1 and Cy5 on X0-4 (Supplementary Table S2). Reaction was incubated without protein in the presence of 20 mM HEPES (pH 7.5), 2.5 mM MgCl_2_, 100 mM NaCl, 0.1 mM DTT and 0.1 mg ml^–1^ BSA at 37 °C for 15 min with 125 nM DNA substrate. The reaction was initiated with 1 μM protein (*At*-HIGLE, *At*-HIGLE^E95Q^, *At*-HIGLE^1–183^ and *At*-HIGL^E184-368^). Aliquots were taken out at different time points, and the reaction was quenched using 5.0 mM EDTA followed by 2 mg ml^–1^ proteinase K (NEB), 0.2% SDS treatment of the samples. The samples were run on 10% Native-TBE-PAGE at 150 V (20 V cm^–1^) for 35 min and scanned using Typhoon scanner. Quantification was done using Image Quant software (GE Healthcare).

Mapping of the cleavage sites by *At*-HIGLE and *At*-HIGLE^1–183^ on different DNA substrates was carried out by resolving the products (after 90 min reaction) on 12% TBE-Urea polyacrylamide gel at a constant voltage of 100 V (15 V cm^–1^) for 2 h. The samples were prepared by heating the reaction mixture for 10 min at 95 °C in formamide containing Orange G dye. The cleavages in 6-FAM and Cy5 labeled DNA strands were monitored independently by scanning the gels for 6-FAM and Cy5 signals using Typhoon scanner. The incision sites were mapped by using 6-FAM and Cy5 labeled DNA marker oligonucleotides of different lengths. The markers used for mapping the cleavage sites were chemically synthesized followed by HPLC and gel purification. Sequence of the oligonucleotides used as the marker is derived from the DNA oligos 6-FAM labeled X0-1 (X0-1^f^) and Cy5 labeled X0-4 (X0-4^c^) (Supplementary Table S2C).

### Cruciform assays

Plasmid pIRbke8^mut^ (23,60) was transformed into TOP10 competent cells, and colonies were selected on LB plates containing 100 μg ml^–1^ ampicillin. Plasmid DNA was isolated using Qiagen Midiprep Kit. For each experiment, plasmid DNA was diluted to 10 nM with water. Reaction was performed with 0.5 nM plasmid in a buffer containing 50 mM Tris (pH 8.5), 2.5 mM MgCl_2_, 1.0 mM DTT, 100 mM NaCl and 0.1 mg ml^–^^1^ BSA and incubated at 37 °C for 30 min to induce cruciform extrusion. Cleavage reaction was then initiated by adding 100 nM protein. Aliquots were taken out at different time points, and the reaction was quenched using 5.0 mM EDTA followed by 2 mg ml^–1^ proteinase K (NEB), 0.2% SDS treatment of the samples. Products were analyzed on 0.8% agarose gel stained with SYBR Gold (Invitrogen™) for 2 h and visualized under Gel Doc XR+ System (Bio-Rad).

### Fluorescence anisotropy

The binding of *At-*HIGLE and its variants with various branched DNA substrates were studied using fluorescence anisotropy. DNA substrates were labeled with 6-FAM on F0-1. (Supplementary Table S3). Substrates were used at a concentration of 25 nM (15 nM labeled and 10 nM unlabeled), and protein concentration ranged from 0 to 600 nM. Reaction was set up in a buffer containing 20 mM HEPES (pH 7.5), 2.5 mM CaCl_2_, 100 mM NaCl, 0.5 mM DTT, 2 mM EDTA and 0.1 mg ml^–1^ BSA at 25°C. Binding reactions were set in Corning 96 flat bottom black polystyrene plates. Anisotropy was measured using a POLARstar Omega microplate reader at an excitation wavelength of 485 nm and an emission wavelength of 520 nm. Fluorescence anisotropy was calculated as (*I*_||_ – *I*_⊥_)/(*I*_||_ + 2*I*_⊥_), where *I*_||_ and *I*_⊥_ are intensities in parallel and perpendicular directions. Binding was studied as the change in anisotropy (*A* – *A*_0_) versus protein concentration, where *A* is the observed anisotropy, and *A*_0_ is the anisotropy of substrate alone. All experiments were done in triplicate. Dissociation constant (*K*_d_) was calculated as (*A* – *A*_0_) = amplitude*[protein]/([protein] + *K*_d_).

### Oligomeric state determination

Size exclusion chromatography (SEC) was performed to analyze the oligomeric state of *At-*HIGLE, *At*-HIGLE^1–183^ and *At*-HIGLE^184-368^ using the AKTA-FPLC system (Amersham). Sephacryl 16/60 S-100 (for *At*-HIGLE^1–183^ and *At*-HIGLE^184–368^) and Sephacryl 16/60 S-200 columns (for *At*-HIGLE) were equilibrated with 20 mM Tris–HCl (pH 8.5), 5% glycerol, 5 mM beta-mercaptoethanol and 0.5 M NaCl. The column was calibrated using gel filtration markers (Bio-RAD). A standard curve was generated using gel filtration markers (Vitamin B12, Myoglobin, Ovalbumin and gamma globulin). Kav was calculated as (*V*e – *V*o)/(*V*t – *V*o), where *V*e, *V*o and *V*t are elution volume, void volume, and total volume, respectively. Thyroglobulin (670 kDa) was used to determine the column's void volume.

### Circular dichroism

Circular dichroism (CD) experiments were conducted on a Jasco J-815 spectropolarimeter with a Peltier-type temperature controller (Jasco CDF-426 S/15). The far-UV CD spectra of *At*-HIGLE and its mutants were measured in the wavelength range 260–195 nm using a quartz cuvette with 0.1 cm path length. The data points were recorded using scan speed 100 nm min^–1^ and spectral bandwidth 1.0 nm with 25 accumulations per sample. A protein concentration of 0.3 mg ml^–1^ in 20 mM Tris–HCl (pH 8.5), 100 mM NaCl, 5% glycerol and 5 mM β-mercaptoethanol was used for all the measurements. Buffer contribution was subtracted from all protein spectra.

## RESULTS

### GIY-YIG endonuclease superfamily: plants *vs*. other organisms

**Figure 1.**
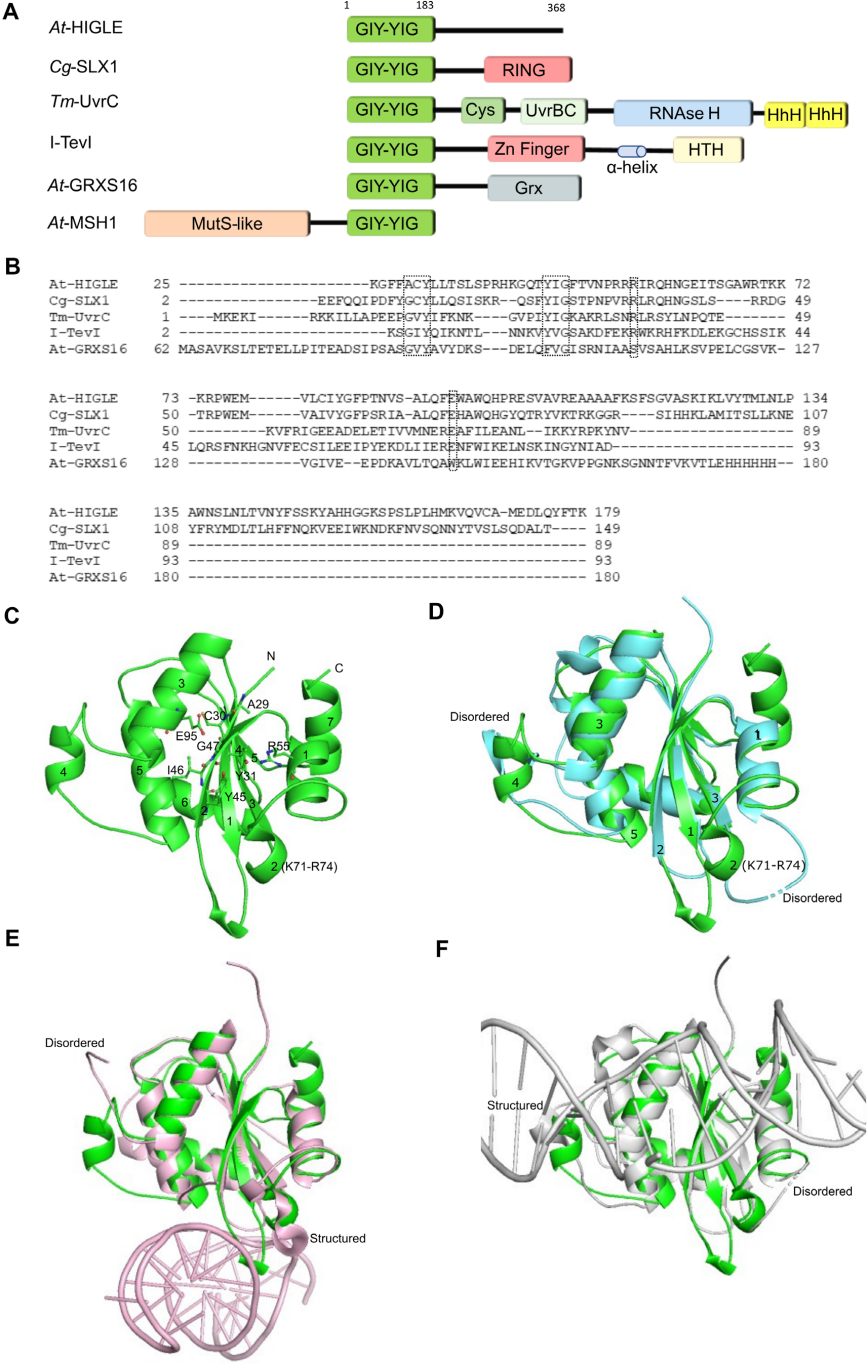
The overall structure of *At*-HIGLE. (**A**) Domain architecture of selected GIY-YIG endonuclease members. The dark green block indicates the GIY-YIG domain. RING: Really Interesting New Gene Zinc finger motif; Cys: conserved Cys residues rich region; UvrBC: UvrB interacting domain; RNAse H: Ribonuclease H domain; HhH: helix-hairpin-helix motif; HTH: helix-turn-helix domain; Grx: Glutaredoxin module. (**B**) Structure-based sequence alignment of GIY-YIG domains of *At*-HIGLE (from *Arabidopsis thaliana*), *Cg*-SLX1 (Structure-selective endonuclease from *Candida glabrata*; PDB: 4XM5), *Tm*-UvrC (nucleotide excision repair protein from *Thermotoga maritima*; PDB: 1YCZ), I-Tev1 (homing endonuclease from Enterobacteria phage T4; PDB: 1LN0), and *At*-GRXS16 (glutaredoxin from *A. thaliana*; PDB: 2LWF). GIY-YIG motif, conserved Arg residue, and metal chelating conserved Glu residues are highlighted. (**C**) The overall structure of GIY-YIG domain of *At*-HIGLE. All the signature amino acid residues of the GIY-YIG motif are shown in the sticks. Secondary structure features are numbered. (**D**) Superimposition of GIY-YIG domains of *At*-HIGLE (green) and *Cg*-SLX1 nuclease domain (cyan; PDB: 4XM5) highlighting the differences between the two proteins. The loops disordered in *Cg*-SLX1 structure are demarcated. (**E**) Superimposition of GIY-YIG domains of *At*-HIGLE (green) and DNA bound *Tt*-SLX1 (nuclease domain) (Pink) (PDB: 6SEI). (**F**) Superimposition of GIY-YIG domains of *At*-HIGLE (green) and DNA bound *Sc*-SLX1 (nuclease domain) (grey) (PDB: 7CQ4).

The hallmark of the GIY-YIG endonuclease superfamily is a signature GIY-YIG hairpin followed by conserved Arg and Glu residues. The GIY-YIG nucleases are often associated with additional domains (Figure 1A). These accessory domains regulate the catalytic activity and govern the substrate specificity of the associated GIY-YIG nuclease domain. The GIY-YIG domain of UvrC generates a 3′ incision in a damaged DNA with the help of a UvrB interacting region and tandem HhH motif at the C-terminal end (61,62). I-TevI homing endonuclease from bacteriophage T4 contains a sequence-specific DNA interacting C-terminal region (63). SLX1 from animals and fungi comprises an N-terminal GIY-YIG nuclease domain and a C-terminal RING domain. The RING domain of fungal SLX1 regulates nuclease activity by participating in SLX1 homodimerization and interacting with SLX4 (40) (Figure 1A). SLX1 in complex with SLX4 is a key HJ resolving nuclease in fungi and animals. A plant protein similar in structure and function to fungal and animal SLX1 is unknown. A yeast two-hybrid screening led to the identification of a novel GIY-YIG containing endonuclease, *At*-HIGLE, from *A. thaliana* (51).

*At*-HIGLE comprises an N-terminal GIY-YIG nuclease domain (amino acid residues 1–183) and an extended C-terminal region (amino acid residues 184-368) (Figure 1A). Structure-based sequence alignment showed that the nuclease domain of *At*-HIGLE has conserved residues in the framework region with the signature hairpin GIY-YIG motif modified to ACY-YIG. The hairpin is followed by conserved Arginine (Arg55) and metal ion chelating Glutamate (Glu95) residues (Figure 1B). The C-terminal region of *At*-HIGLE does not show similarity with any other known protein fold. Although, the N-terminal domain of *At*-HIGLE aligns with a high sequence similarity with the nuclease domains of SLX1 proteins, there is no sequence similarity between the extended C-terminal region of *At*-HIGLE and the RING domain of SLX1 from animals and fungi (Supplementary Figure S2). The other characterized GIY-YIG containing plant proteins include a mismatch-repair protein (MSH1: Muts homolog 1) (64) and a glutaredoxin (GRXS16) (65) (Figure 1). Therefore, we decided to explore *At*-HIGLE structurally and biochemically to scrutinize its potential function in HJ resolution in order to confirm whether *At*-HIGLE is indeed the hitherto unreported SLX1 from plants.

### Overall structure of *At*-HIGLE

Full-length *At*-HIGLE was subjected to extensive crystallization trials to unravel its structural organization. Eventually, *At*-HIGLE was truncated (*At*-HIGLE^1–183^) based on secondary structure predictions to remove disordered segments at the C-terminal end (Supplementary Figure S3). *At*-HIGLE^1–183^ crystallized in *P*2_1_2_1_2_1_ space group with one molecule in the asymmetric unit. Crystals diffracted to a resolution of 1.7 Å. Phases were determined using molecular replacement with SLX1 from *Candida glabrata* (*Cg*-SLX1) as the starting model (PDB: 4XM5) (40). The structure was refined to an R_free_ of 19.38% (Table 1, Supplementary Figure S4). The structure exhibits a mixed α/β topology. *At*-HIGLE^1–183^ comprises of a β sheet formed by five β strands oriented as β_2_–β_1_–β_3_–β_4_–β_5_ (Figure 1C, Supplementary Figure S5) with β_2_ and β_3_ strands present in anti-parallel orientations with respect to β_1_, β_4_ and β_5_ strands. While helices α_1_, α_2_ and α_7_ are present on one side of the β sheet, helices α_3_–α_6_ are present on the other side. Poor electron densities did not allow the modeling of amino acids 1–26 at the N-terminal end and 180–183 at the C-terminal end. Comparison of the *At*-HIGLE structure with other members of the GIY-YIG endonuclease superfamily clearly showed that *At-*HIGLE is the most similar to SLX1 and different from other members of the GIY-YIG family. *At*-HIGLE^1–183^ superimposes with the nuclease domain of *Cg*-SLX1 with a root-mean square deviation (r.m.s.d.) of 0.8 Å (Figure 1D, Supplementary Figure S6). Furthermore, a DALI (66) search using the nuclease domain of *At*-HIGLE, identified SLX1 proteins (PDBs: 6SEI, 6SEH, 7CQ2, 7CQ3, 4XLG and 4XM5) to be the closest hits with a Z-score of above 15.0. The Z-score with UvrC protein in the DALI search (PDB: 1YD6) is 3.5. Therefore, the structure confirms *At*-HIGLE to be a member of the SLX1 family of GIY-YIG nuclease superfamily.

Compared to the nuclease domain of *Cg*-Slx1, *At*-HIGLE^1–183^ showed differences in the conformation of loops relevant to DNA substrate binding. Loop connecting α_1_ and β_3_ is unmodelled (and thus presumably flexible) in apo structures of SLX1: *Cg*-SLX1 (PDB: 4XM5), *Cg*-SLX1-SLX4^CCD^ (PDB: 4XLG) and *Tt*-SLX1-SLX4^CCD^ (complex from *Thermothielavioides terrestris*) (PDB: 6SEH), while it adopts a one-turn helix (K71-R74) in case of *At*-HIGLE, a conformation adopted in DNA bound structure of *Tt*-SLX1-SLX4^CCD^ (PDB: 6SEI) (Figure 1D, Figure 1E, Supplementary Figure S7). Likewise, the loop connecting α_3_ and α_5_ of *At*-HIGLE adopts a helical conformation (named as α_4_) (Figure 1C, D). In contrast, the corresponding loops (i.e. between α_2_ and α_3_) in *Cg*-SLX1 (PDB: 4XM5), *Cg*-SLX1-SLX4^CCD^ (PDB: 4XLG) and *Tt*-SLX1-SLX4^CCD^ (PDB: 6SEH) are unstructured. Interestingly, the same loop in DNA bound structure of *Sc*-SLX1-SLX4^SAP+CCD^ (complex from *Saccharomyces cerevisiae*) (PDB: 7CQ4) (67) adopts a conformation similar to *At*-HIGLE (Figure 1F, Supplementary Figure S7). Therefore, *At*-HIGLE has substrate interacting loops in conformations ready for DNA binding. The average *B*-factors for the loop connecting α_1_ and β_3_, and the loop connecting α_3_ and α_5_ are 21.62 and 18.32 Å^2^, respectively, whereas the average B-factors for the structure is 18.57 Å^2^ indicating stable conformation of the loops. A third loop, connecting β1 and β2 (Figure 1D), is longer in the case of *At*-HIGLE than *Cg*-SLX1, and because of its proximity to one of the DNA binding sites, it may exert influence on protein-DNA interactions. In summary, *At*-HIGLE exists in a conformation ready for interactions with DNA substrate.

### Catalytic activity of *At*-HIGLE

**Figure 2.**
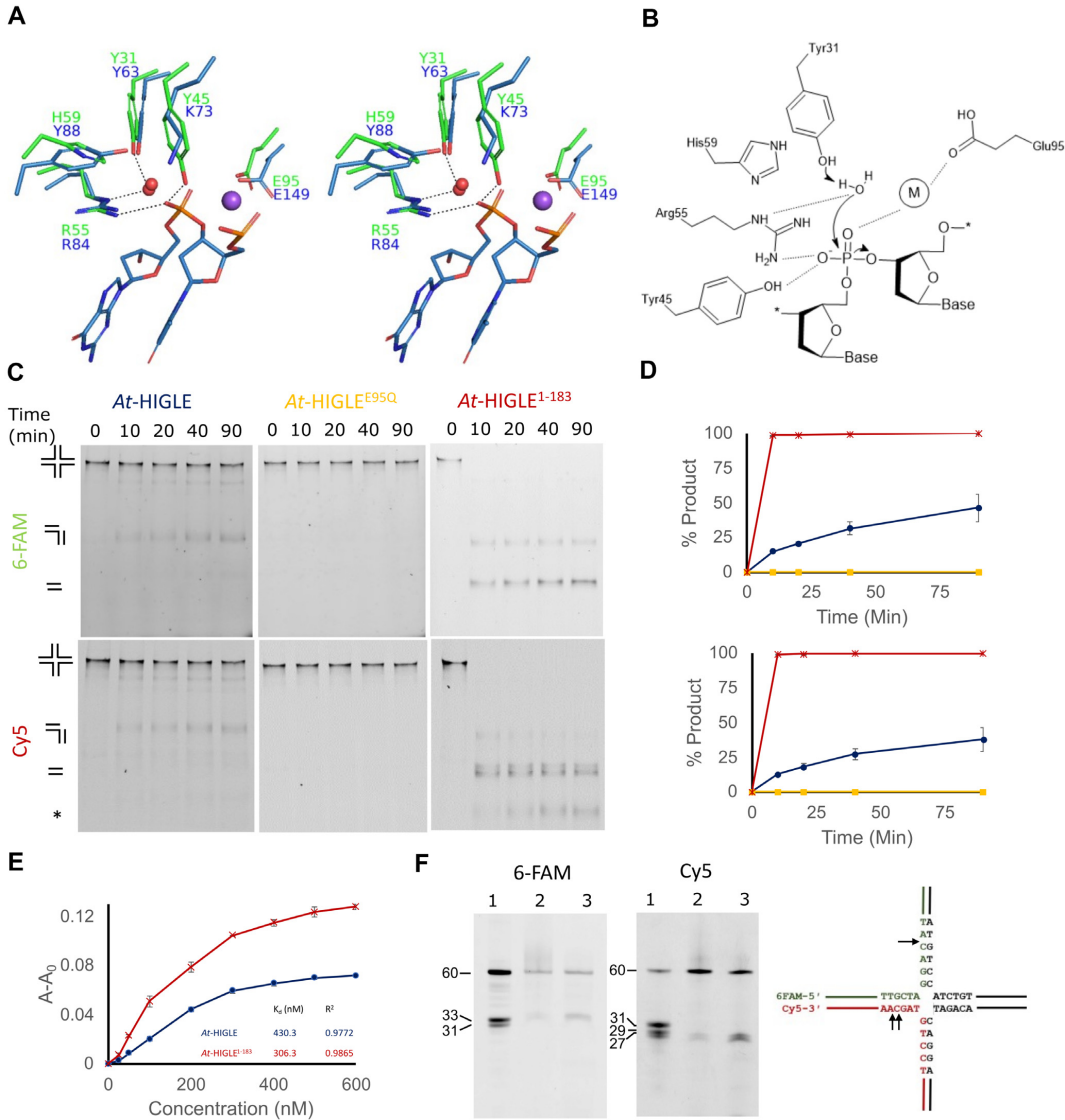
Catalytic activity of *At*-HIGLE on Holliday Junction. (**A**) Superimposition of the active sites of *At*-HIGLE (green) and Hpy188I restriction endonuclease (Blue) (PDB: 3OQG). Water molecules and sodium ion are shown in red and purple colors, respectively. Hydrogen bonds are shown as dashed lines. (**B**) A schematic of the possible catalytic mechanism. The metal ion is labeled as M. (**C**) Catalytic activity of *At*-HIGLE on a synthetic Holliday Junction (HJ). Two of the four DNA strands are labeled with 6-FAM (at 5′ end) and Cy5 (at 3′ end). The reaction products were resolved on a 10% native TBE-PAGE. Upper panels: gels scanned for 6-FAM signal; lower panels: gels scanned for Cy5 signal. Left gels: *At*-HIGLE (full length); middle gels (*At-*HIGLE^E95Q^, catalytically inactive mutant); right gels: *At*-HIGLE^1–183^. The schematic of the predicted products during nuclease action have been shown. The asterisk represents an undefined product. All the experiments were done in triplicate. (**D**) Quantitation of the product after catalytic activity of *At*-HIGLE on an HJ with standard error bars. Upper panel: quantification for 6-FAM signal; lower panel: quantification for Cy5 signal. *At*-HIGLE (full length), *At*-HIGLE^E95Q^, and *At-*HIGLE^1–183^ are shown in blue, yellow, and red colors, respectively. **(E)** Holliday Junction binding studies. Binding studies with Holliday Junction in the presence of *At*-HIGLE (full length) (blue) and *At*-HIGLE^1–183^ (red) using fluorescence anisotropy with standard error bars. Signal for 6-FAM was used for anisotropy experiments. Y-axis is shown as change in anisotropy (*A* – *A*_0_), where *A* is observed anisotropy and *A*_0_ is anisotropy of DNA substrate alone. All experiments were done in triplicates. (**F**) Mapping of the cleavage site. The reaction mixture from 90 min of reaction with *At*-HIGLE (full length) and *At*-HIGLE^1–183^ were resolved on a 12% TBE urea–PAGE. Left panel: signal for 6-FAM; right panel: signal for Cy5. Lane1: ladder; lane 2: reaction with *At*-HIGLE (Full length); lane 3: reaction with *At*-HIGLE^1–183^.

Crystal structures of Hpy188I and R.Eco29kI restriction endonucleases provide comprehensive insights into the catalytic mechanism of GIY-YIG endonuclease superfamily (68,69). A structural comparison of *At*-HIGLE with Hpy188I restriction endonuclease provides mechanistic insights into the catalysis by *At*-HIGLE (Figure 2A). *At*-HIGLE has a catalytic site typical of GIY-YIG endonucleases. During hydrolysis of the phosphodiester bond, a water molecule or a hydroxide performs a nucleophilic attack from one side, and a metal ion interacts with the scissile phosphate from the other side to facilitate a single substitution reaction. The metal ion destabilizes the substrate and stabilizes the transition state. Catalytically essential amino acid residues are Glu95, Arg55, Tyr31 and Tyr45. Glu95 chelates a metal ion, whereas Arg55 and Tyr31 coordinate the attacking nucleophile. Arg55 and Tyr45 interact with the scissile phosphate (Figure 2B). In the absence of a direct experimental evidence for the catalytic mechanism of GIY-YIG nucleases, possibility of phosphodiester bond hydrolysis through a two-step mechanism involving a phosphotyrosine intermediate cannot be ruled out (70).

A highly conserved catalytic site and a close structural similarity with SLX1 encouraged us to investigate the catalytic activity of *At*-HIGLE on fluorescently labeled synthetic Holliday Junctions (Figure 2C and 2D, Supplementary Figures S8 and S9). The substrate was prepared by annealing four different strands. Of the four DNA strands, two were fluorescently labeled; one with 6-FAM at the 5′ end and a second with Cy5 at the 3′ end (Supplementary Table S2). The rationale of using two fluorescent labels is to monitor cleavage in different DNA strands of synthetic DNA substrates. *At*-HIGLE and *At*-HIGLE^1–183^ were active on HJ substrate, which showed that the enzyme does not require any additional regulatory protein for activation. A variant with a substitution of metal-chelating Glu95 to Gln (*At*-HIGLE^E95Q^) was catalytically inactive, which confirmed the observed activity was inherent to *At*-HIGLE and not to any potential contaminating nucleases (Figure 2 and Supplementary Figure S9). Therefore, *At*-HIGLE can cleave branched DNAs (HJs), but unlike fungal and animal SLX1, it does not require any accessory protein, SLX4, for its catalytic activity.

In our activity assays, we also tested the truncated variant (*At*-HIGLE^1–183^) . Remarkably, the activity of the truncated variant was much higher than the full-length protein. Moreover, the full-length protein does not immediately proceed with the secondary reaction after the initial nick (i.e. nicked or gapped DNA is not processed further). Although, a possibility of secondary reaction with full-length *At*-HIGLE at later time-points cannot be ruled out. In contrast, *At*-HIGLE^1–183^ continues to cleave nicked DNA (or gapped DNA), as evident from two sets of products on native TBE-PAGE (Figure 2C). Therefore, the C-terminal domain of *At-*HIGLE reduces its catalytic activity. Interestingly, fluorescence anisotropy measurements showed that *At*-HIGLE^1–183^ and full-length protein have comparable binding affinities for HJ substrate (Figure 2E). Collectively, these results indicate that the C-terminal region regulates the activity of *At*-HIGLE.

Eventually, we analyzed the reactions from the 90 min time points from the activity assays (Figure 2C) on a denaturing PAGE to map the sites of cleavages (Figure 2F). No differences were observed in the cleavage pattern of Holliday Junctions by the full-length and truncated *At*-HIGLE. Both full-length and truncated *At*-HIGLE cleaved 6-FAM as well as Cy5 labeled DNA strands. The cleavage sites are located three-four nucleotides from the crossover point toward the 3′ side (Figure 2F), a characteristic common to SLX1 proteins from fungi and animals (26,27,38–40,71,72).

### *At*-HIGLE activity on multiple branched DNA molecules

**Figure 3.**
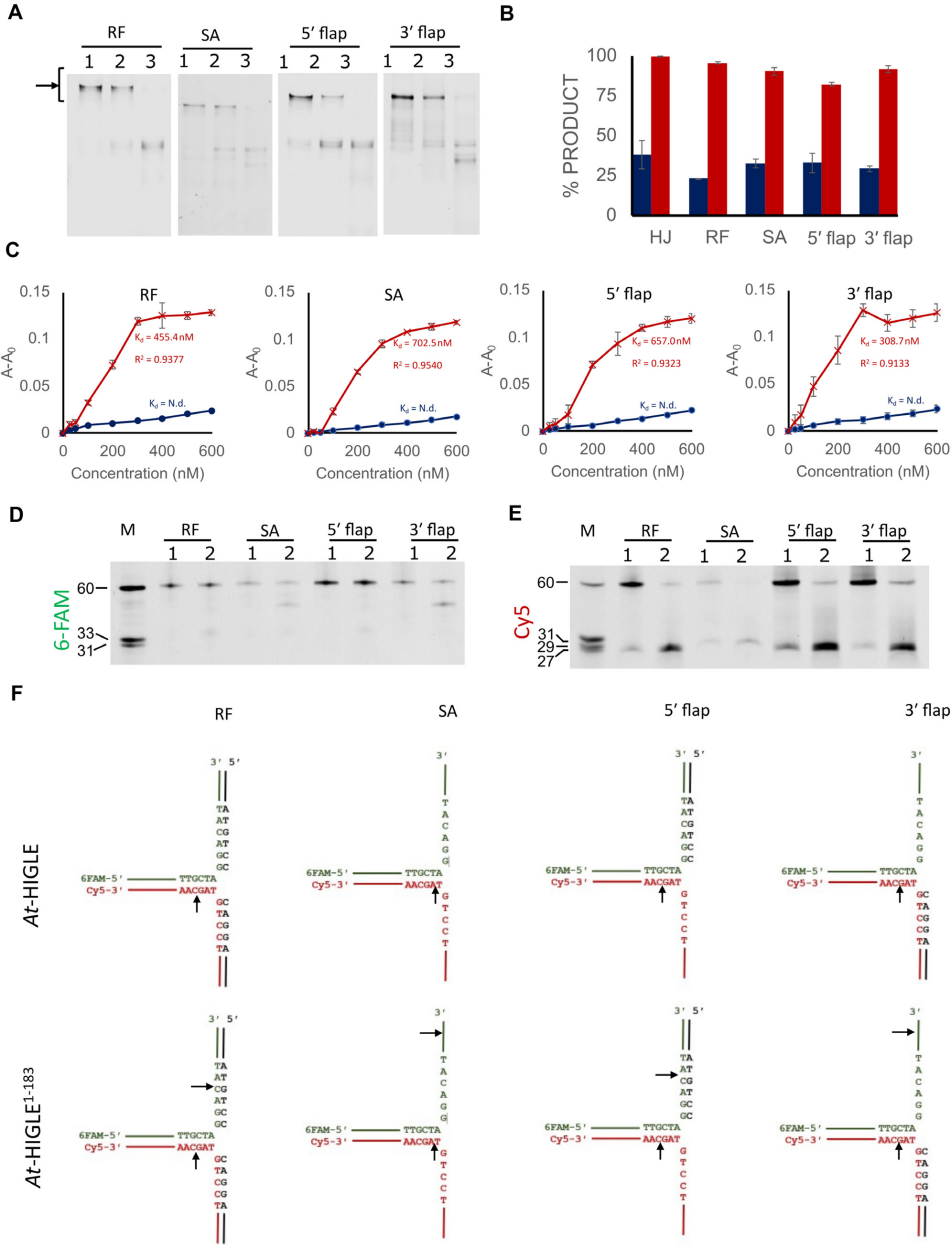
Substrate specificity of *At*-HIGLE. (**A**) The catalytic activity of *At*-HIGLE (full-length protein) (lane 2) and N-terminal domain of *At*-HIGLE (*At*-HIGLE^1–183^) (lane 3) on various joint DNA molecules: replication fork (RF), 5′ flap, 3′ flap, and splayed arm (SA). Lane 1 is substrate alone. Reaction on Cy5 labeled DNA strand was followed. The reaction products were resolved on a 10% native TBE-PAGE. Substrates are shown with an arrow. (**B**) Quantitation of product formed with standard error bars after 90 min reaction. Reaction with full length (*At*-HIGLE) and *At*-HIGLE^1–183^ are shown in blue and red colors, respectively. Values for reaction with Holliday Junction (HJ) are used from the experiment reported in Figure 2 for comparison. All the experiments were done in triplicates. (**C**) Binding studies with joint DNA molecules in the presence of *At*-HIGLE (full length) (blue) and *At*-HIGLE^1–183^ (red) using fluorescence anisotropy with standard error bars. Signal for 6-FAM was used for anisotropy experiments. Y-axis is shown as change in anisotropy (A-A_0_), where A is observed anisotropy and A_0_ is anisotropy of DNA substrate alone. Protein concentration is plotted on X-axis. All experiments were done in triplicates. N.d. denotes K_d_ values not determined. (**D**) The reaction mixture from 90 minutes of reaction with *At*-HIGLE (full length) (lane 1) and *At*-HIGLE^1–183^ (lane 2) were resolved on a 12% TBE Urea-PAGE to follow the cleavage of 6-FAM labeled DNA strand. (**E**) The reaction mixture from 90 minutes of reaction with *At-*HIGLE (full length) (lane 1) and *At*-HIGLE^1–183^ (lane 2) were resolved on a 12% TBE Urea-PAGE to follow the cleavage of Cy5 labeled DNA strand. (**F**) Mapping of the cleavage sites. Cleavage sites resulting from the activities of *At*-HIGLE (full length) (upper panel) and *At*-HIGLE^1–183^ (lower panel) are shown with black arrows.

The unique feature of SLX1 among GIY-YIG superfamily endonucleases, in general, is its promiscuous substrate specificity – the ability to cleave various branched DNA substrates. A close structural similarity of *At*-HIGLE with SLX1 prompted us to test the catalytic activity of *At*-HIGLE on other branched DNA substrates. Similar to SLX1, *At*-HIGLE could cleave a variety of branched DNA molecules: replication fork, 5′ flap, 3′ flap, and splayed arm substrates (Figure 3A, Supplementary Figure S10). Consistent with the results with HJ (Figure 2), full-length *At*-HIGLE was less active than *At*-HIGLE^1–183^ on all tested branched DNA substrates (Figure 3B). The increased catalytic activity of the truncated variant was in line with its higher affinity for the tested branched DNA substrates (Figure 3C).

We next mapped the cleavage sites for each joint DNA molecules using the full-length *At*-HIGLE and *At*-HIGLE^1–183^. *At*-HIGLE generates nick only in the Cy5 labeled DNA strand and no cleavage in 6-FAM labeled strand. The cleavage occurs three nucleotides from the junction point in the 3′ direction in the cases of RF, 5′ flap, and 3′ flap. In the case of splayed arm substrate, cleavage occurs one nucleotide from the junction point in the 3′ direction. Interestingly, *At*-HIGLE^1–183^ can generate nicks in both 6-FAM and Cy5 labeled DNA strands. The nicks made by *At*-HIGLE^1–183^ on 6-FAM labeled strands are also toward the 3′ end from the junction point. In the cases of 3′ flap and splayed arm substrates, nicks in 6-FAM labeled strand are far from the junction point, implying non-specific binding of the single-stranded portion of these two substrates near the catalytic site of *At*-HIGLE^1–183^ (Figure 3D–F). Both *At*-HIGLE and *At*-HIGLE^1–183^ bind single-stranded DNA. However, only *At*-HIGLE^1–183^ can generate nick in the single-stranded DNA towards the 3′ edge as seen in the cleavage patterns of 3′ flap and splayed-arm substrates (Supplementary Figure S11). In summary, all branched DNA substrates are cut in similar sites, and *At*-HIGLE^1–183^ could introduce additional nicks in branched DNA substrates.

### Model of DNA binding

**Figure 4.**
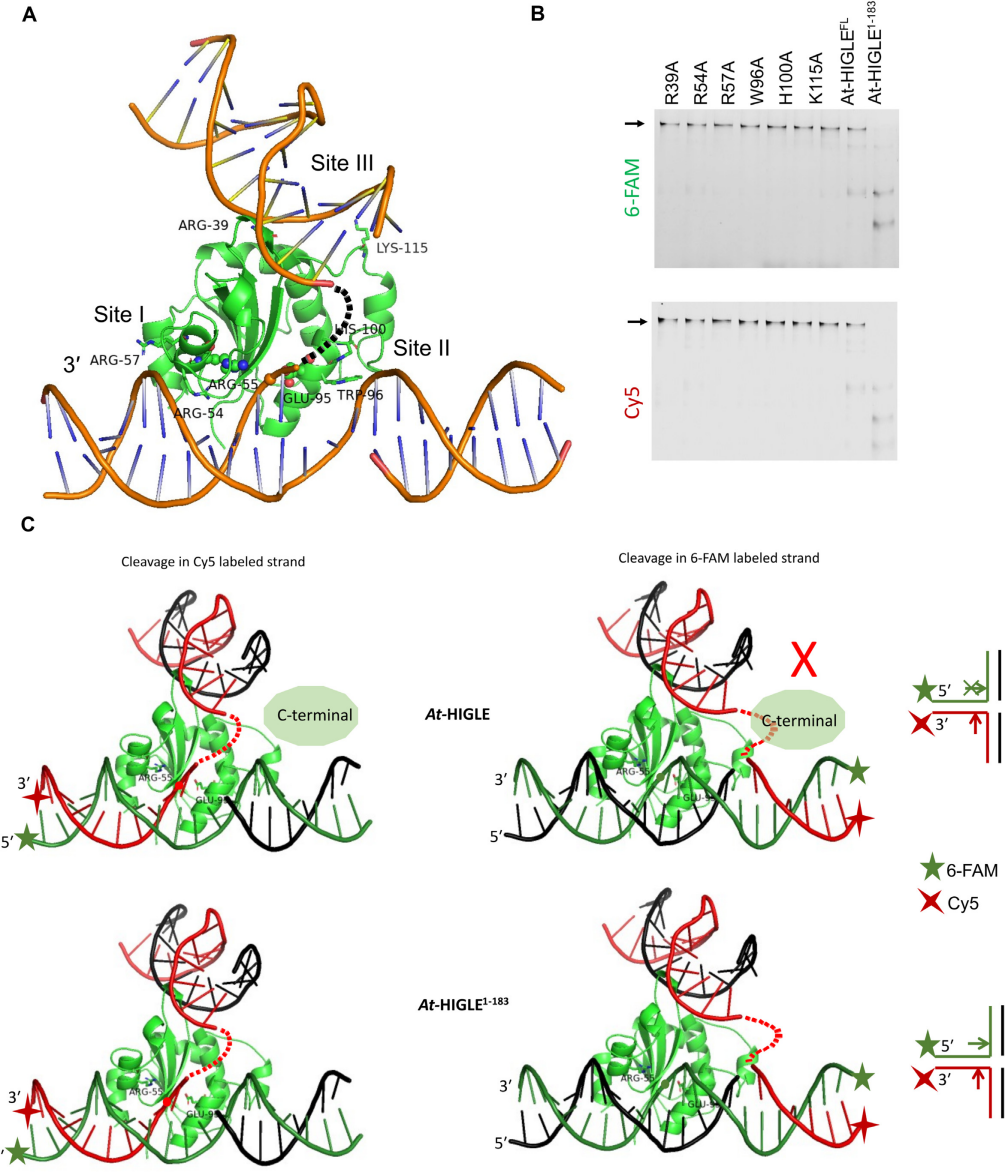
DNA binding model. (**A**) A DNA binding model depicting catalytic domain of *At*-HIGLE interacting with a replication fork. The bound DNA was modeled by superimposing catalytic domain of *At*-HIGLE with DNA bound structure of R.Eco29kI (PDB: 3NIC) and DNA bound structure of *Thielavia terrestris* Slx1-Slx4^CCD3^ complex (PDB: 6SEI). The 3′ end of the DNA strand undergoing cleavage has been highlighted. Two catalytically important active site residues: Arg55 and Glu95, are shown as balls and sticks. Amino acid residues predicted to interact with various branches of DNA substrates are shown as sticks. The scissile phosphate is shown with a sphere. (**B**) The catalytic activity of DNA binding mutants on a synthetic HJ labeled with 6-FAM and Cy5. *At*-HIGLE (WT) was used as a positive control. The first lane in the gel contains no protein. The gel was scanned for both 6-FAM and Cy5 signals. (**C**) Models explaining cleavages in Cy5 and 6-FAM labeled strands of a replication fork. While *At*-HIGLE^1–183^ can make nicks in both strands, *At*-HIGLE (full length) can only make nick in Cy5 labeled strand. Multiple nicks by *At*-HIGLE^1–183^ are possible only because of substrate's multiple binding mode, which is predicted to be restricted by steric hindrance from the C-terminal region in the case of full length *At*-HIGLE as depicted by cartoon. The scissile phosphate in 6-FAM labeled and Cy5 labeled DNA strands are shown with green and red circles respectively.

Two DNA-bound structures of SLX1 are currently available: (1) *Tt*-Slx1-Slx4^CCD^ in complex with a hairpin-like DNA (PDB: 6SEI) (39) and (2) *Sc*-Slx1-Slx4^SAP+CCD^ in complex with 5′ flap DNA substrate (PDB: 7CQ4) (67). In none of these structures, the DNA substrate is bound to the enzyme in a catalytic configuration. DNA bound structures of R.Eco29kI and Hpy188I provide insights into the alignment of DNA substrate around the catalytic site of GIY-YIG endonucleases (68,69). The insights into the catalytic interaction of DNA with GIY-YIG nuclease are provided by the structure of a substrate complex of R.Eco29kI (PDB: 3NIC). This structure provides information about the orientations of the DNA strand that undergoes cleavage and the non-cleaved strand around the active site. This enables the identification of two DNA binding sites: site I and site II (Figure 4A). DNA-bound structure of *Tt*-Slx1-Slx4^CCD^ revealed a third interface for DNA interaction (site III) away from the active site (39) (Figure 4A). The DNA-bound structure of *Sc*-Slx1-Slx4^SAP+CCD^ contains a 5′flap substrate with a local distortion at the active site induced by the single nucleotide flap being wedged into the DNA duplex (67). Nevertheless, the structure confirmed the role of site I and site II in binding the two arms of a branched DNA substrate. If extended, the single nucleotide flap can reach and bind positively charged amino acid residues at site III. Further confirmation of DNA binding by site III comes from the interaction of a symmetry-related DNA molecule with this region in the structure of *Sc*-Slx1-Slx4^SAP+CCD^ (PDB: 7CQ4). Therefore, based on the DNA bound structures of R.Eco29kI (PDB: 3NIC), *Tt*-Slx1-Slx4^CCD^ (PDB: 6SEI) and *Sc*-Slx1-Slx4^SAP+CCD^ (PDB: 7CQ4), we generated a DNA substrate-binding model for *At*-HIGLE to understand the interaction of various arms of branched DNA substrates (Figure 4A, Supplementary Figures S12 and S13).

The *At*-HIGLE-DNA substrate model allowed us to identify amino acid residues involved in DNA substrate binding. These included Arg57 and Arg54 from site I, Trp96 and His100 from site II, and Arg39 and Lys115 from site III (Figure 4A). The DNA-binding residues of *At*-HIGLE identified based on the model are strictly conserved among plants (Supplementary Figure S5). The site I plays a vital role in orienting the DNA substrates in the correct register at the active site. Arg57 and Arg54 interact with the non-cleaved DNA strand. This interaction allows the complementary cleavable strand to enter the active site with Arg55 on one side and the metal-chelating Glu95 on the other side, enabling an in-line nucleophilic attack. DNA duplex bound at site III is at a sharp angle relative to DNA bound at site I resulting in bending in the DNA substrate near the branch point. Site II is involved in interacting with the remaining arm of the DNA substrate. We validated the model by testing the effects of substituting the residues predicted to bind the DNA. The tested variants included R39A, R54A, R57A, W96A, H100A and K115A. The overall protein structure of mutants was assessed with circular dichroism spectroscopy with some deviations observed in the cases of R54A and K115A mutants (Supplementary Figure S14). All tested variants lost most of their catalytic activity and had greatly reduced affinity for DNA (Figure 4B, Supplementary Figure S14). Moreover, our model agrees with the observed cleavage site in the DNA strand located toward the 3′ side from the junction point (Figures 2 and 3). Therefore, biochemical results corroborated with the proposed model of *At*-HIGLE interacting with branched DNA.

### Binding and processing of branched DNA substrates

The binding of branched DNA substrates and the nicking pattern is a complex interplay between the three DNA-binding sites (Sites I, II and III). *At*-HIGLE cleaves an HJ by generating nicks in 6-FAM and Cy5 labeled DNA strands (Figure 2). The activity of *At*-HIGLE on an HJ is, therefore, identical to *Cg*-Slx1-Slx4^CCD^. However, the main difference appears in the processing of non-symmetrical branched-DNA molecules (Replication fork, 5′ flap, 3′ flap, and splayed arm substrate) by *At*-HIGLE. Full length *At*-HIGLE specifically cleaves Cy5 labeled strand of non-symmetrical branched DNA substrates with no activity on 6-FAM labeled strand (Figure 3). In contrast, *Cg*-Slx1-Slx4^CCD^ can cleave both strands (39). Multiple modes of branched DNA substrate binding utilizing the three DNA binding sites explain the activity of *Cg*-Slx1-Slx4^CCD^ on both the stands (39). Processing of only one DNA strand by *At*-HIGLE indicates it prefers to bind non-symmetrical branched DNA substrates only in one orientation (Figure 4C, SI Figure 13). The processing of branched DNA substrate's arm with no discontinuity at the junction point is favored for catalysis. This distinction is absent in the case of HJs because of the molecule's symmetrical nature. The binding of asymmetrical joint DNA molecules in one particular orientation is a unique feature of *At*-HIGLE compared to SLX1.

A truncated *At*-HIGLE (*At*-HIGLE^1–183^) can process both the DNA strands (6-FAM and Cy5 labeled) of symmetric and non-symmetric branched DNA substrates (Figure 2 and 3). It is, therefore, evident that the C-terminal region of *At*-HIGLE restricts the conformational space in which a branched DNA substrate can interact with *At*-HIGLE. The C-terminal region can regulate the conformational space of DNA binding either through sterically clashing with branched DNA substrates in specific orientations (Figure 4C and Supplementary Figure S13) or by directly interacting with the DNA substrate. Attempts were made to express the C-terminal region of *At*-HIGLE (*At*-HIGLE^184–368^) (Supplementary Figures S15 and S16) alone and assess its DNA binding potential. However, most of the protein was produced in aggregated form, and we could not get sufficient material for DNA binding studies. Although the role of the *At*-HIGLE^184–368^ in interaction with branched DNA substrate cannot be ruled out, the steric hindrance by the C-terminal region of *At*-HIGLE appears to be a more plausible scenario since truncation of the C-terminal region increases the binding affinity to all the DNA substrates, increases the catalytic activity and allows the enzyme to cleave both 6-FAM and Cy5 labeled DNA strands (Figures 2 and 3).

### Holliday Junction resolution and the oligomeric state of *At*-HIGLE

**Figure 5.**
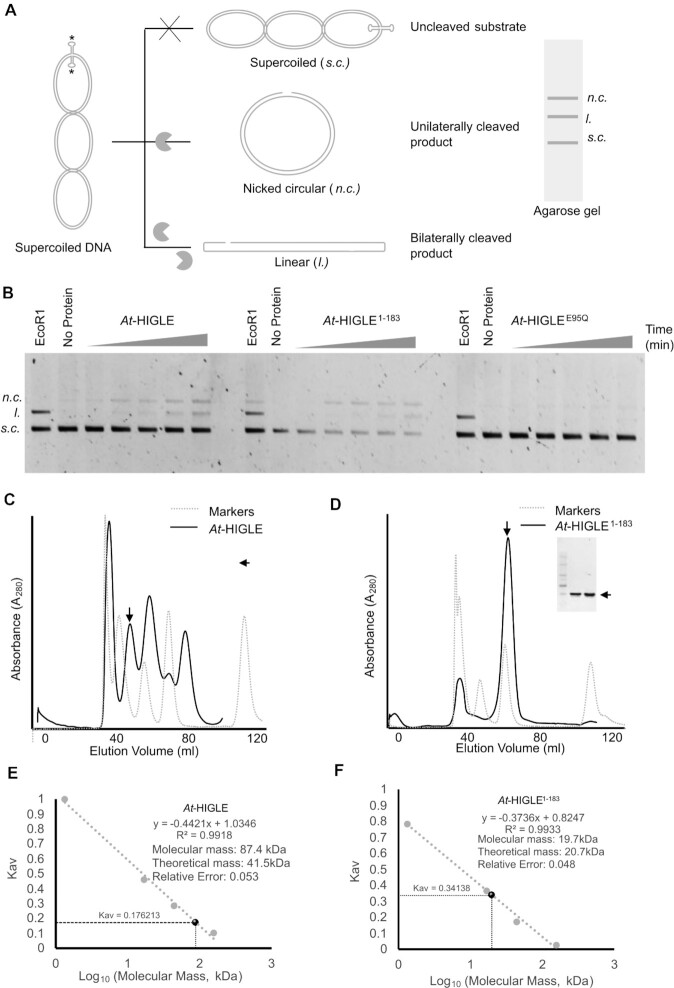
Resolution of Holliday Junction and the oligomeric state of *At*-HIGLE. (**A**) A schematic of cruciform nuclease assay using a pIRbke8^mut^ plasmid containing an inverted repeat sequence that adopts a conformation mimicking a Holliday Junction. Asterisks represent the sites for EcoRI restriction endonuclease. The plasmid is purified in a supercoiled state (*s.c*.). Single nick results in the release of supercoils resulting in a nicked circular plasmid (*n.c*.), whereas two simultaneous nicks result in a linear product (*l*.) formation. (**B**) Time-dependent cruciform assays using *At*-HIGLE (full-length) (Left gel), *At*-HIGLE^1–183^ (middle gel), and *At*-HIGLE^E95Q^ (inactive mutant, full-length) (Right gel). EcoRI serves as a positive control generating a linear product. The second lane in each gel is substrate alone. All the reactions were run on 0.8% Agarose gel. (**C**) Size-exclusion chromatogram (black) of *At*-HIGLE purified on a Sephacryl S200 gel filtration column superimposed with gel filtration markers (broken grey). The peak corresponding to *At*-HIGLE is demarcated by an arrow. The fractions from the demarcated peak were run on a 12% SDS-PAGE. (**D**) Size-exclusion chromatogram (black) of *At*-HIGLE^1–183^ purified on a Sephacryl S100 gel filtration column superimposed with gel filtration markers (broken grey). The peak corresponding to *At*-HIGLE^1–183^ is demarcated by an arrow. The fractions from the demarcated peak were run on a 12% SDS-PAGE. (**E**) Estimation of the oligomeric weight of *At*-HIGLE from a standard curve generated using gel filtration markers (Vitamin B12, Myoglobin, Ovalbumin, and gamma globulin (**F**) Estimation of the oligomeric weight of *At*-HIGLE^1–183^ from a standard curve generated using gel filtration markers (Vitamin B12, Myoglobin, Ovalbumin, and gamma globulin). Kav was calculated as (*V*e – *V*o)/(*V*t - Vo) where *V*e, *V*o and *V*t are elution volume, void volume and total volume, respectively. Thyroglobulin (670 kDa) was used to determine the column's void volume.

The complete HJ resolution requires the introduction of two nicks across the Holliday Junction. The first incision is rate-limiting followed by a second coordinated incision within the life-time of the protein-DNA complex. To study the ability of *At*-HIGLE to perform a HJ resolution we used a cruciform assay on a supercoiled plasmid (pIRbke8^mut^) with a cruciform-like structure (Figure 5). In a cruciform assay, a single nick on the supercoiled plasmid (pIRbke8^mut^) results in the resorption of cruciform and the formation of a nicked circular plasmid. On the other hand, two nicks within the life-time of protein–DNA complex result in a linear plasmid (Figure 5A). A cruciform assay established the HJ resolution potential of *At*-GEN1 and *At*-SEND1 (46) similar to human GEN1 (73). GEN1 exists as a monomer in the solution and dimerizes on binding an HJ, facilitating two nicks and eventually resolving an HJ (73,74).

The full-length *At*-HIGLE generates both linear and nicked-circular plasmids. The appearance of a linear plasmid suggested that *At*-HIGLE has the potential to perform two incisions within the lifetime of the protein-DNA complex. The appearance of nicked circular plasmid provides clues about the possible non-canonical activity of *At*-HIGLE, similar to Yen1, which has both canonical and non-canonical activities (75) (Figure 5B). Since, *At*-HIGLE has a potential to generate two incisions within the life-time of a single protein–DNA complex, there are two possible scenarios: either *At*-HIGLE dimerizes on interacting with an HJ as in the case of human GEN1 (73,74), or *At*-HIGLE exists as a homodimer in the solution as in the case of RuvC (19,76). Therefore, we determined the oligomeric state of full-length *At*-HIGLE and *At*-HIGLE^1–183^ in the solution (Figure 5C–F, Supplementary Figure S17). Full-length *At*-HIGLE exists as a homodimer in solution. This implies that *At*-HIGLE might function as a canonical resolvase—a dimeric protein introducing two nicks into an HJ by utilizing two active sites within the dimer. *At*-HIGLE^1–183^ exists as a monomer in the solution, and generates a nicked-circular plasmid in the cruciform assay with linear plasmid appearing during the later time-point in the reaction. The presence of full-length *At*-HIGLE in a dimeric state and *At*-HIGLE^1–183^ in monomeric state points toward the involvement of the C-terminal region of *At*-HIGLE in governing the oligomeric state of the protein. In fact, the C-terminal region of *At*-HIGLE (*At*-HIGLE^184–368^) exists as a dimer in the solution (Supplementary Figure S15) and has no catalytic activity (Supplementary Figure S16).

## DISCUSSION

Regulation of the structure-selective endonucleases is essential for the maintenance of genome stability (77,78). For instance, G2/M arrest of HIV-infected cells results from an untimely activation of SLX4, leading to enhanced cleavage of DNA by MUS81–EME1 complex (79). Therefore, SSEs are under tight regulation: to be activated at the right time at the right place (8). SSEs regulation operates at different levels: cell cycle, nuclear localization, post-translational modification, and protein-protein interaction. Nuclear exclusion of GEN1 until nuclear envelope breaks limits its activity on HJs (80,81). Cell cycle-dependent phosphorylation of MUS81–EME1 (G2/M transition) increases its activity (81). SLX1 relies on SLX4 for its catalytic activity. Besides, SLX4 couples the catalytic activities of SLX1 and MUS81–EME1 during HJ resolution (36). SLX4 undergoes CDK1 dependent phosphorylation that plays a role in MUS81–SLX4 interaction (82).

Fungal SLX1 exists as a homodimer with inaccessible DNA binding and catalytic residues. An interaction of SLX1 with the CCD (C-terminal conserved domain) domain of SLX4 disrupts this inhibitory homodimer and exposes the catalytic site and DNA binding residues (39,40). Therefore, SLX4 is the crucial regulator of SLX1, which is otherwise a highly promiscuous endonuclease. Our structural and biochemical studies establish the *At*-HIGLE of *A. thaliana* is being equivalent to SLX1 from fungi and animals. Similar to SLX1, *At*-HIGLE also exists as a homodimer in solution. However, unlike fungal SLX1, the *At*-HIGLE homodimer is catalytically active. While catalytic activity of many structure-specific endonucleases depends upon oligomerization of the protein, e.g. RuvC (19,76), GEN1 (73,74), SLX1 (39,40) and *At*-HIGLE, oligomerization driven nuclease activity is also identified in the cases of other nucleases, e.g. restriction endonucleases (68), transposases (83), Drosha-DGCR8 (84), SARS2 Nsp15 nuclease (85). While in fungi, SLX4 regulates the catalytic activity of SLX1, in the case of *At*-HIGLE, the C-terminal region regulates its own catalytic activity. The regulation by the C-terminal domain of *At*-HIGLE is mediated by moderating the substrate-binding, and modulating the rate of catalysis. Interestingly, based on secondary structure predictions and Alphafold2 (86), the C-terminal domain of *At*-HIGLE is predicted to be largely devoid of any defined structure. However, involvement of C-terminal domain in an interaction with the branched DNA substrate cannot be ruled out. Although a protein similar to SLX4 is unknown in plants, *At*-HIGLE has an inbuilt mechanism to regulate its own catalytic activity. Removal of the C-terminal region releases this regulatory mechanism resulting in vigorous catalysis by the *At*-HIGLE.

The substrate specificity of SSEs is dependent mainly upon their specific structural attributes. Although RuvC shows preference toward a particular sequence at the crossover point (5′-A/T TT↓G/C-3′) (18), most SSEs are specific for the structure adopted by branched DNA molecules. GEN1 dimerizes on binding an intact HJ, undergoes a transition of its active site from disordered to ordered state, and bends a DNA substrate with the help of a wedge and an H2TH motif (Helix-two-turn-helix) (23,74). Conformational changes undergone by MUS81-EME1 on binding DNA substrate followed by the formation of a 5′phosphate binding pocket dictates its substrate preference for nicked HJ over an intact HJ (33,34). SLX1 and *At*-HIGLE comprise surface-exposed positively charged residues organized as distinct patches (Supplementary Figure S18). These positively charged patches interact with various arms of branched DNA substrates. Therefore, SLX1 and *At*-HIGLE can bind and process HJ, RF, 3′ flap, 5′ flap and splayed arms with varying degrees of efficiency. Furthermore, SLX1 and *At*-HIGLE identify the branching point in the DNA substrate by locating structural discontinuity in DNA by the spatial arrangement of DNA binding patches at an angle with each other (39,40).

*At*-HIGLE generates nick specifically in the double-stranded portion of the arm without any discontinuity at the junction. In contrast, SLX1 can generate incisions in all the arms of branched DNA substrates making *At*-HIGLE a more specific SSE than SLX1. This distinction between *At*-HIGLE and SLX1 disappears with the truncation of the C-terminal region of *At*-HIGLE. The C-terminal region of *At*-HIGLE is indeed guiding the conformational space in which a branched DNA substrate can interact with *At*-HIGLE. The conformational space of DNA substrate binding is further restricted by already ordered DNA binding loops of *At*-HIGLE as observed in the crystal structure of the nuclease domain of *At*-HIGLE. In the cases of *Cg-*SLX1 and *Tt*-SLX1, the DNA binding sites are primarily disordered and undergo a transition to an ordered state on interaction with DNA. Altogether, we determined the first structure of SLX1 lineage member of GIY-YIG nuclease superfamily from the plant kingdom and established its role in HJ resolution. Our biochemical data provide novel insights into an in-built mechanism that regulates the catalytic activity of *At*-HIGLE.

Among humans, Holliday Junctions are processed through dissolution involving BTR complex (BLM helicase-TOPOIIIα-RMI1-RMI2) or resolution involving SSEs (also called resolvases). Processing of HJs through BTR complex is the preferred mechanism in mammalian mitotic cells. Mutations in BLM helicase are associated with Bloom's syndrome characterized by chromosomal instability, immunodeficiency, and an early onset of cancer conditions (87). Bloom's syndrome cells exhibit increased resolvases mediated sister chromatid exchange (88,89). The phenotype can be suppressed by depleting resolvases in Bloom's syndrome cells, indicating dissolution and resolution are parallel pathways. The combined deletion of MUS81 and SLX1 have a phenotype similar to the single deletion of either MUS81 or SLX1, suggesting the two proteins work in the same pathway. However, depletion of GEN1 either with SLX1 or MUS81 has an additive effect, indicating GEN1 operates in a pathway independent of SLX1 and MUS81 (89). Furthermore, the SLX1–SLX4 complex interacts with MUS81–EME1 during the G2/M phase of the cell cycle. The catalytic sites of the two nucleases cooperate to resolve HJ through a nick-and-counter-nick mechanism. The interaction between SLX1 and MUS81-EME1 is mediated through SLX4 (36). SLX4 is a multidomain scaffolding protein interacting with several nucleases. Mutations in SLX4 are associated with a recessive human disorder, Fanconi anemia (25–28,41,42).

**Figure 6.**
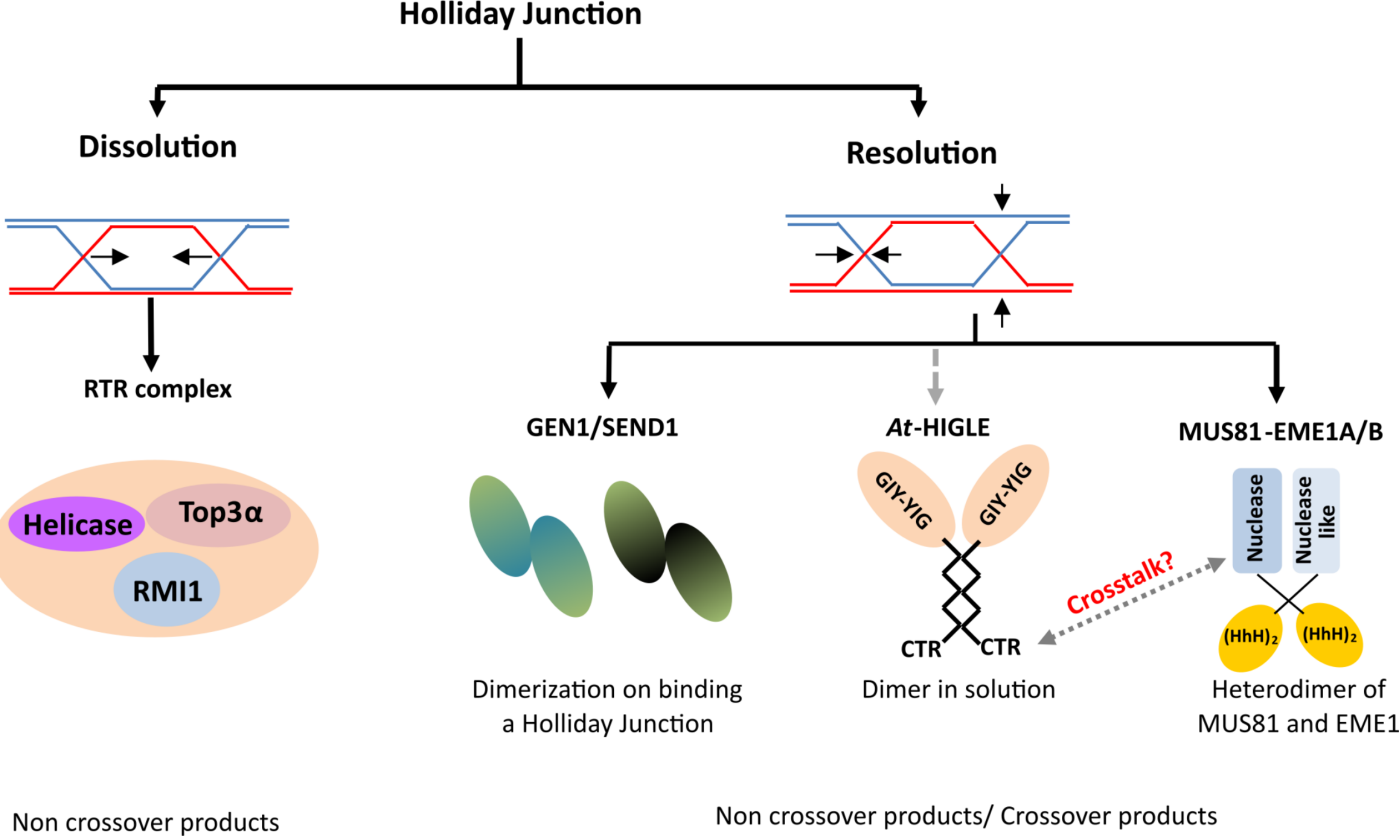
Processing of Holliday junction in plants. Dissolution by an RTR complex (RecQ Helicase/TOP3α/RMI1) results in non-crossover product formation. Resolution of Holliday Junctions is carried out by canonical endonucleases (GEN1 and SEND1) and non-canonical endonuclease (MUS81-EME1). *At*-HIGLE is a GIY-YIG endonuclease similar to fungi and animal SLX1 that can resolve HJ. *At*-HIGLE does not require a protein similar to SLX4 for its activity.

In plants, the entire mechanism of HJ processing is not worked out as extensively as in animals and fungi, with important gaps in our understanding of this process (Figure 6). Among plants, the processing of HJ proceeds through either dissolution or resolution. Dissolution is carried out by an RTR complex (RecQ helicase-Top3α-RMI1) (90–92) while SSEs participate in the resolution. GEN1, SEND1 and MUS81-EME1 are the only plant SSEs known to participate in HJ resolution (44–50,93,94). Similar to animals, emerging evidence suggests that dissolution and resolution are also independent pathways in plants. A mutation in RecQ helicase increases the crossover frequency, and mutation in MUS81 affects meiotic recombination frequency. However, the mutations in both MUS81 and RecQ helicase is lethal among plants (95–98). A disruption in RAD51C (a protein involved in strand invasion and formation of D-loop) can suppress the lethality caused by mutations in RecQ helicase and MUS81 (99). Silencing GEN1 or its loss of function results in male-sterility and persistent double-stranded breaks (94,100). The combined absence of SEND1 and MUS81 results in developmental defects and genome instability (101). Depletion of SEND1 does not enhance the defect caused by an absence of GEN1 (94). Therefore, these accumulated works suggest that GEN1 and SEND1 very likely work in pathways independent of MUS81-EME1.

While MUS81-EME1 from plants acts on intact HJ with reduced efficiency (47), a protein similar to animal SLX1 that can initiate HJ resolution has been unknown. An absence of SLX1 like protein in plants, therefore pose an interesting predicament. In the case of plants, *At-*HIGLE can be one of the SSEs responsible for generating an initial nick for MUS81-EME1. It may also be feasible that *At*-HIGLE might just be acting on its own to resolve HJs because of its dimeric nature. Among animals, SLX4 coordinates the activities of SLX1 and MUS81-EME1 (36). Therefore, it is imperative to determine if the actions of *At*-HIGLE and MUS81-EME1 are coordinate during HJ resolution. Consequently, it is interesting to explore if the C-terminal region of *At*-HIGLE interacts with MUS81-EME1 and its impact on the *At*-HIGLE dimer and catalytic activity. Furthermore, to comprehensively establish the role of *At*-HIGLE in plant homologous recombination and its cross-talk with different components of Holliday Junction processing machinery, the effect of a defect in *At*-HIGLE alone or in combination with *At*-MUS81, *At*-GEN1, *At*-SEND1 and *At*-RecQ4A need to be evaluated through *in vivo* studies, e.g. using homologous recombination assay (102,103).

DNA repair and recombination in plants is a neglected field, especially when many of the pathways have undergone specialization compared to animals and fungi. Deciphering the complete molecular mechanism of HR in plants has dual benefits: (a) understanding the basic biology of DNA repair and recombination in plants, and (b) improvising and innovating genome editing techniques to address the ever-increasing demand for food. One of the major hurdles in any crop improvement program is the low efficiency of crossing over. In principle, an increase in HR frequency has been demonstrated earlier by over-expressing RuvC in plants (104). In-depth knowledge of various pathways operating at the HR level and their cross-talks with other DNA repair and recombination pathways will provide insights to fine-tune the HR frequency in the existing gene-editing techniques for crop improvement in the future.

## Supplementary Material

gkac239_Supplemental_File
