# Supplementary material for: Holliday junction resolution by At-HIGLE: an SLX1 lineage endonuclease from Arabidopsis thaliana with a novel in-built regulatory mechanism
Source: Nucleic Acids Res. 2022 Apr 12;50(8):4630–46. doi: 10.1093/nar/gkac239 (PMC9071465; doi:10.1093/nar/gkac239)
Supplement: gkac239_Supplemental_File [file gkac239_supplemental_file.docx]

**SUPPORTING INFORMATION**

**Holliday Junction resolution by *At*-HIGLE: an SLX1 lineage endonuclease from *Arabidopsis thaliana* with a novel in-built regulatory mechanism.**

**Prabha Verma^1^, Poonam Kumari^1^, Shreya Negi^1^, Gitanjali Yadav^1^, Vineet Gaur^1*^**

1. National Institute of Plant Genome Research, Aruna Asaf Ali Marg, New Delhi, India 110067

^*^Corresponding Author

**Dr. Vineet Gaur**

National Institute of Plant Genome Research

Aruna Asaf Ali Marg

New Delhi, India 110067

[vgaur@nipgr.ac.in](mailto:vgaur@nipgr.ac.in), [vineetgaur1982@gmail.com](mailto:vineetgaur1982@gmail.com)

Ph: +91 26735252


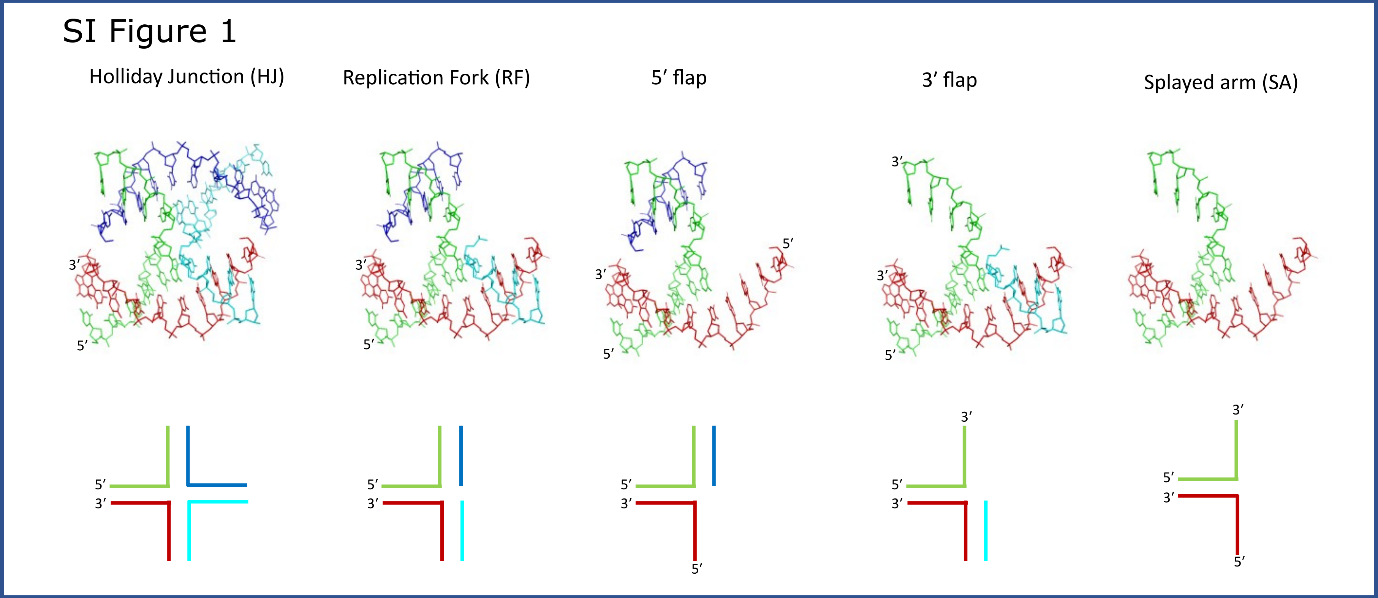


**SI Figure 1. Branched DNA molecules**. various types of branched DNA molecules (also referred to as joint DNA molecules) are often encountered during different processes involving DNA metabolism. Holliday Junction (HJ), Replication forks (RF), 5′ flap, 3′ flap, and splayed arm DNA substrates are shown in stick model (upper panel) along with simplified cartoon (lower panel). PDB: 1DCW was used to generate a figure for the Holliday junction. PDB: 1DCW was edited to create models for the rest of the branched DNA substrates. The structures may not represent the actual conformation of these substrates in the solution.


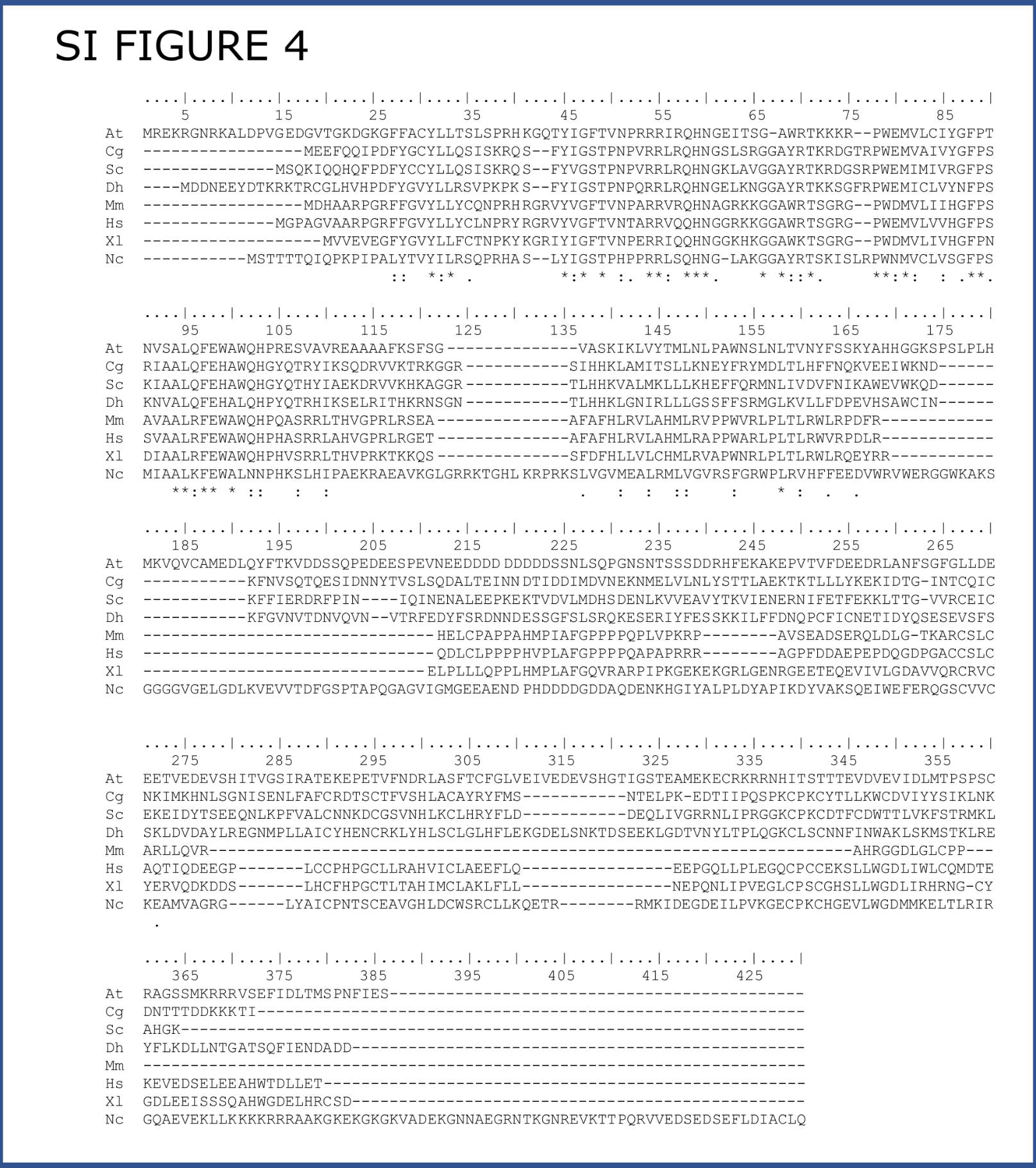


**SI Figure 2. Multiple sequence alignment.** Multiple sequence alignment of representative members of the SLX1 family. The CLUSTAL W Multiple Sequence Alignment Program (version 1.83, Feb 2003) (1). *Arabidopsis thaliana* (At), *Candida glabrata* (Cg), *Saccharomyces cerevisiae* (Sc), *Debaryomyces hansenii* (Dh), *Mus musculus* (Mm), *Homo sapiens* (Hs), *Xenopus laevis* (Xl), *Neurospora crassa* (Nc).

A


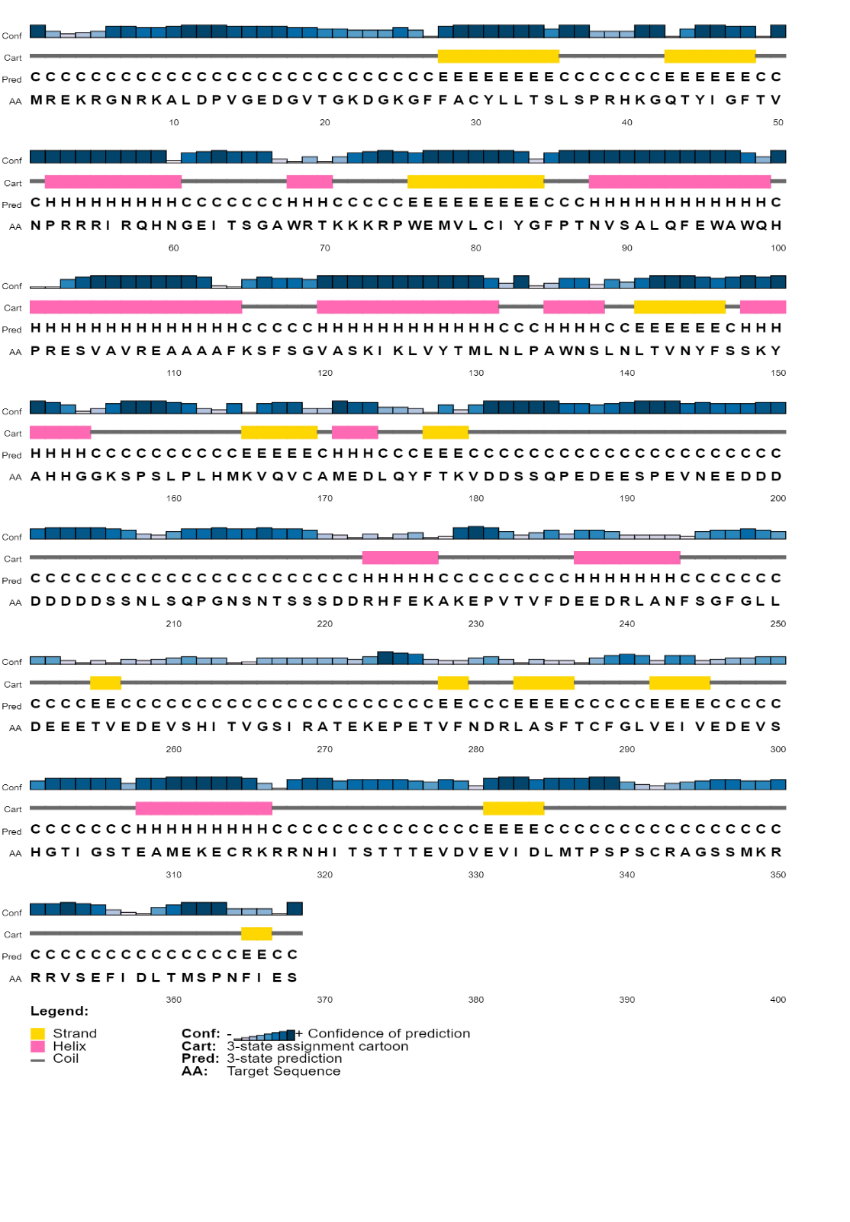


B


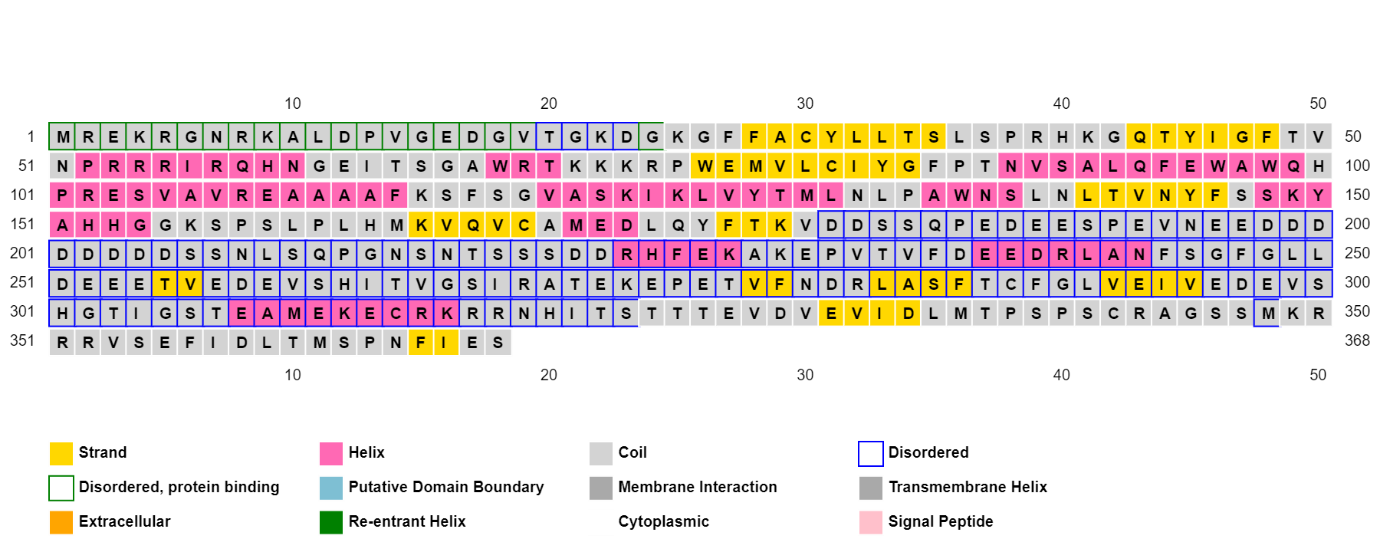


**SI Figure 3. *In silico* analysis of *At*-HIGLE sequence.** **(A)** Secondary structure prediction for *At*-HIGLE. **(B)** Disorder prediction for *At*-HIGLE. Psipred server (<http://bioinf.cs.ucl.ac.uk/psipred/>) was used for secondary structure prediction and disorder prediction (2-4).


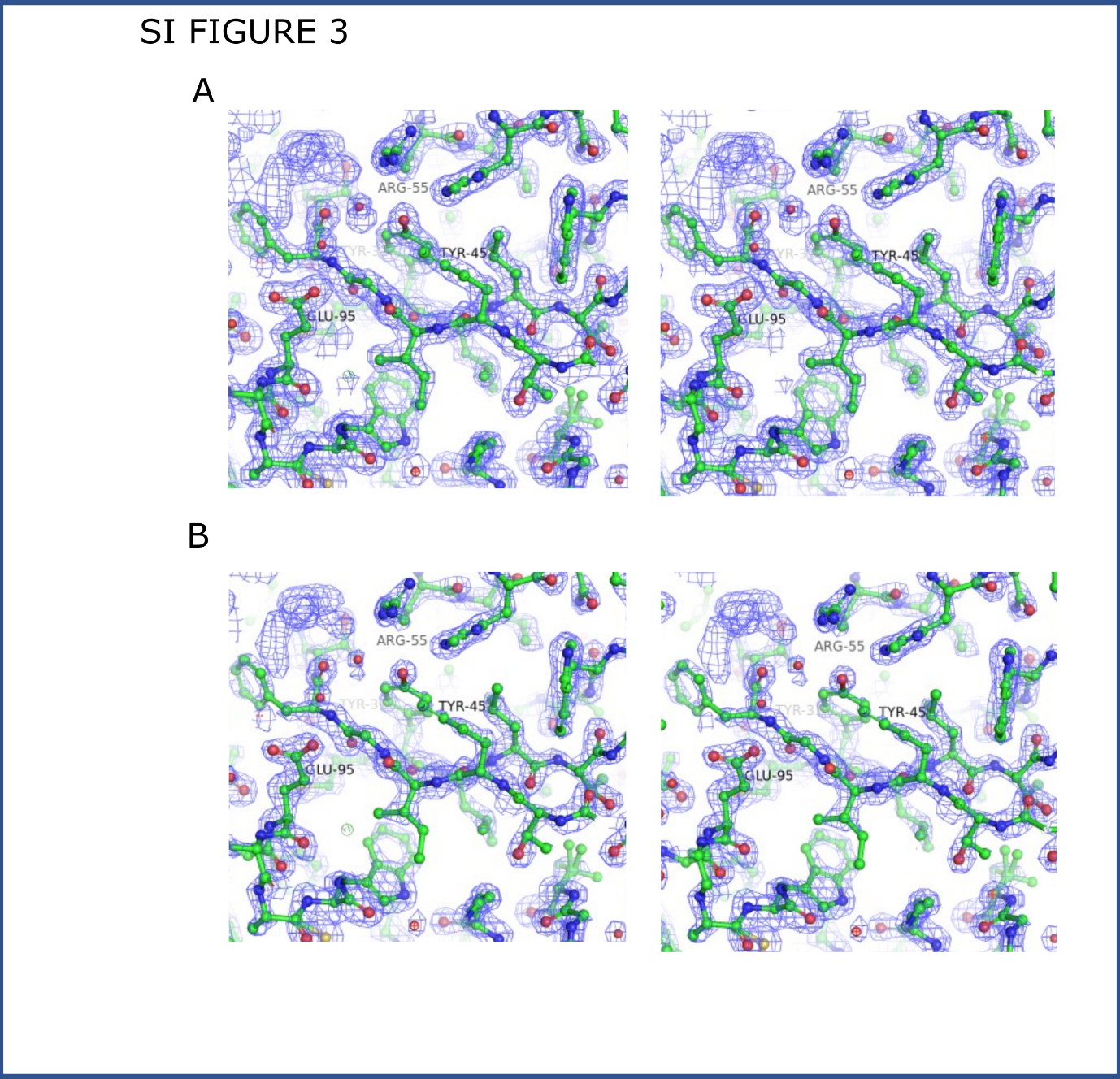


**SI Figure 4. Sample of electron density maps. (A)** Stereoview of 2*F*o-*F*c electron density map (blue mesh) at 2σ along with the ball and stick model of *At*-HIGLE showing active site residues. **(B)** Stereoview of 2*F*o*-F*c simulated annealing composite omit map (blue mesh) at 2σ along with the ball and stick model of *At*-HIGLE showing active site residues.


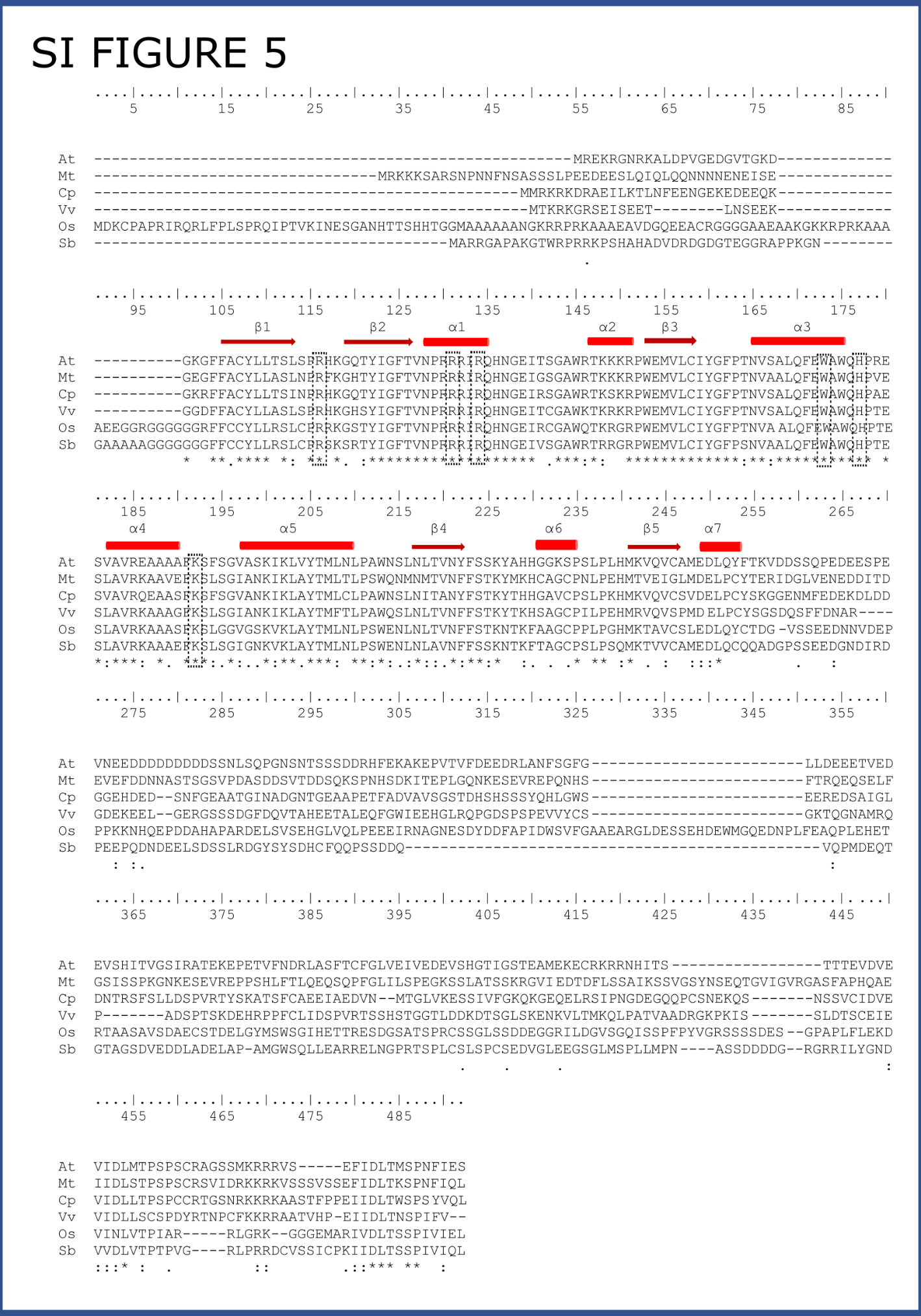


**SI Figure 5. Multiple sequence alignment.** Multiple sequence alignment of *At*-HIGLE with other HIGLE-like proteins from representative plants. The alignment was done using CLUSTAL W Multiple Sequence Alignment Program (version 1.83, Feb 2003) (1). *Arabidopsis thaliana* (At), *Medicago truncatula* (Mt), *Carica papaya* (Cp), *Vitis vinifera* (Vv), *Oryza sativa* (Os), *Sorghum bicolor* (Sb). DNA binding amino acid residues identified based on *At*-HIGLE-DNA substrate model are highlighted with broken boxes.


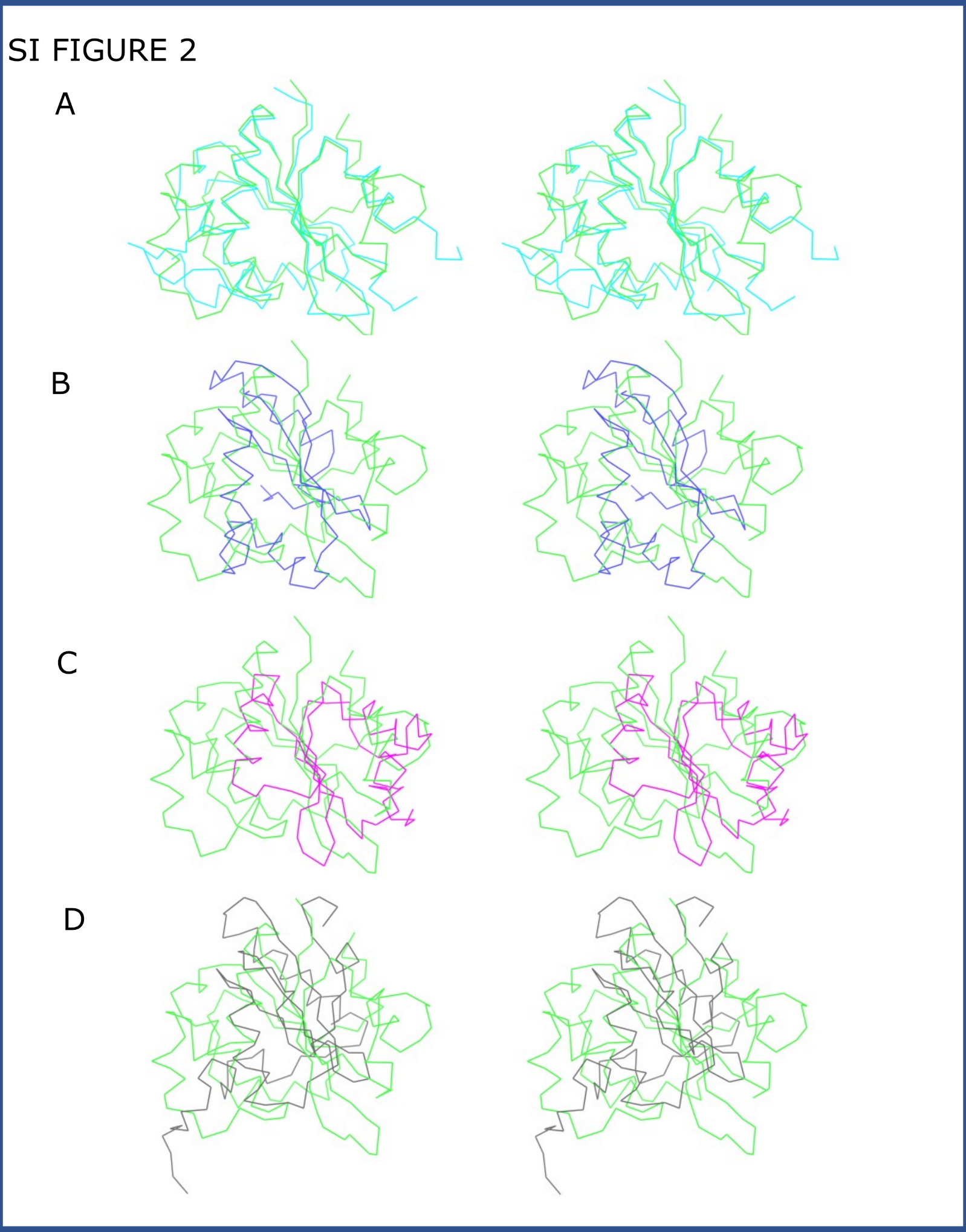


**SI Figure 6.** **Structural comparison of GIY-YIG domains of *At*-HIGLE and selected members of GIY-YIG superfamily in stereo view. (A)** Superimposition of *At*-HIGLE (green) and *Cg*-SLX1 (Cyan; PDB: 4XM5). **(B)** Superimposition of *At*-HIGLE (green) and I-Tev1 (blue; PDB: 1LN0). **(C)** Superimposition of *At*-HIGLE (green) and *Tm*-UvrC (magenta; PDB: 1YCZ). **(D)** Superimposition of *At*-HIGLE (green) and *At*-GRXS16 (gray; PDB: 2LWF).


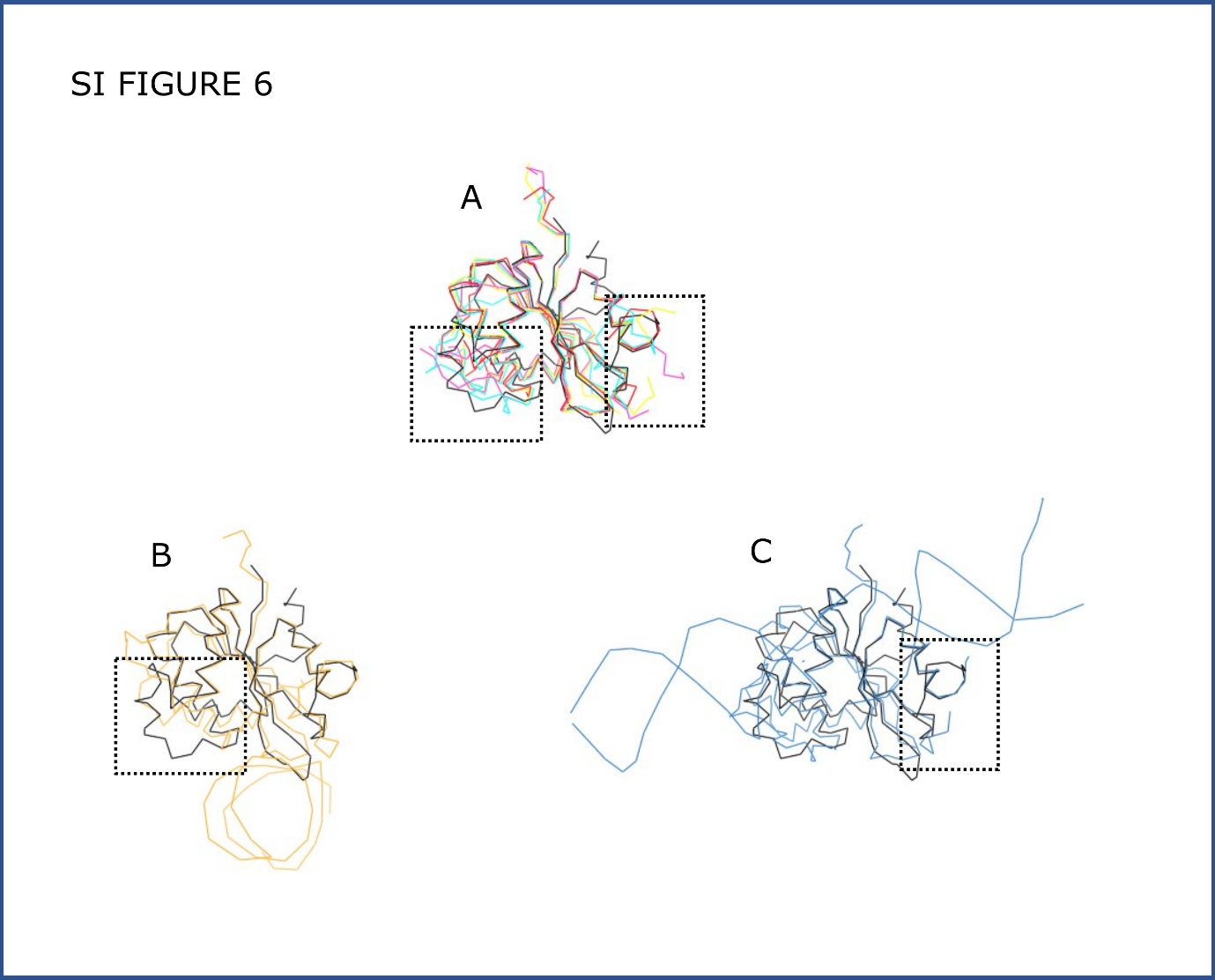


**SI Figure 7. Conformational flexibility in loops important in branched DNA substrate binding.** **(A)** Comparison of the nuclease domains of the apo structures of *At*-HIGLE^1-183^ (black), *Cg*-Slx1 (PDB: 4XLG, yellow), *Cg*-Slx1-Slx4^CCD^ (PDB: 4XM5, magenta), *Tt*-Slx1-Slx4^CCD^ (PDB: 6SEH, red), *Sc*-Slx1-Slx4^SAP+CCD^ (PDB: 7CQ3, cyan). The two flexible loop regions are shown with black boxes. **(B)** Comparison of *At*-HIGLE^1-183^ (black) with the nuclease domain of DNA bound *Tt*-Slx1-Slx4^CCD^ (PDB: 6SEI, orange). The loop adjacent to the bound DNA is present in the same conformation as *At*-HIGLE^1-183^. The other loop is unstructured and depicted by a black box. **(C)** Comparison of *At*-HIGLE^1-183^ (black) with the nuclease domain of DNA bound *Sc*-Slx1-Slx4^SAP+CCD^ (PDB: 7CQ4, blue). The loop adjacent to bound DNA is present in the same conformation as *At*-HIGLE^1-183^. The other loop is unstructured and depicted by a black box.


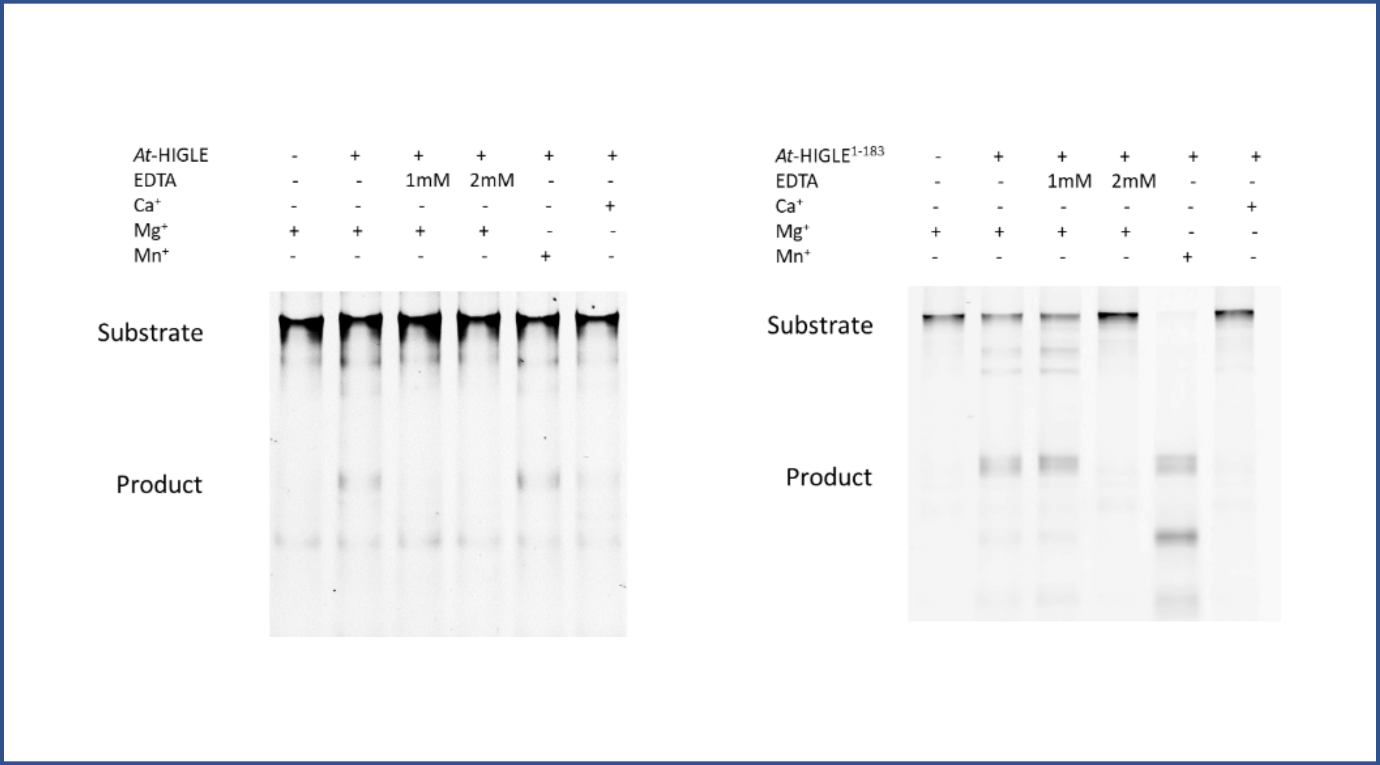


**SI Figure 8. Effect of metal ions and EDTA on *At*-HIGLE and *At*-HIGLE^1-183^ activity on Holliday Junction.** The reactions were carried out with 100nM enzymes, 125 nM DNA substrate (100 nM unlabeled and 25 nM labeled) for 30 min at 37 ⁰C with 1.0 mM metal ion concentration. The gels were scanned for Cy5 signal.


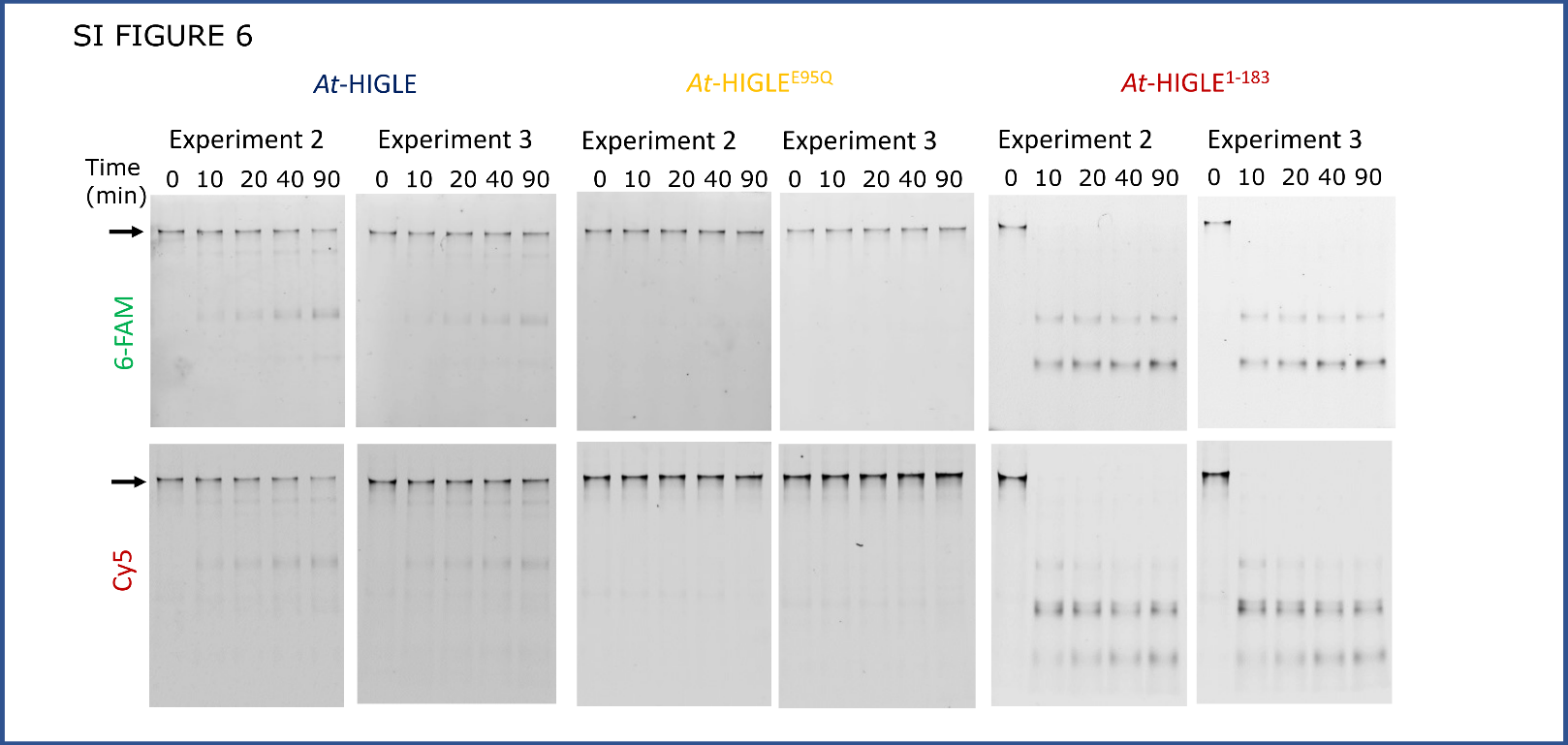


**SI Figure 9. The catalytic activity of *At*-HIGLE on Holliday Junction.** Rest of the activity assays on a synthetic Holliday Junction used for quantitation of product formation reported in Figure 2.


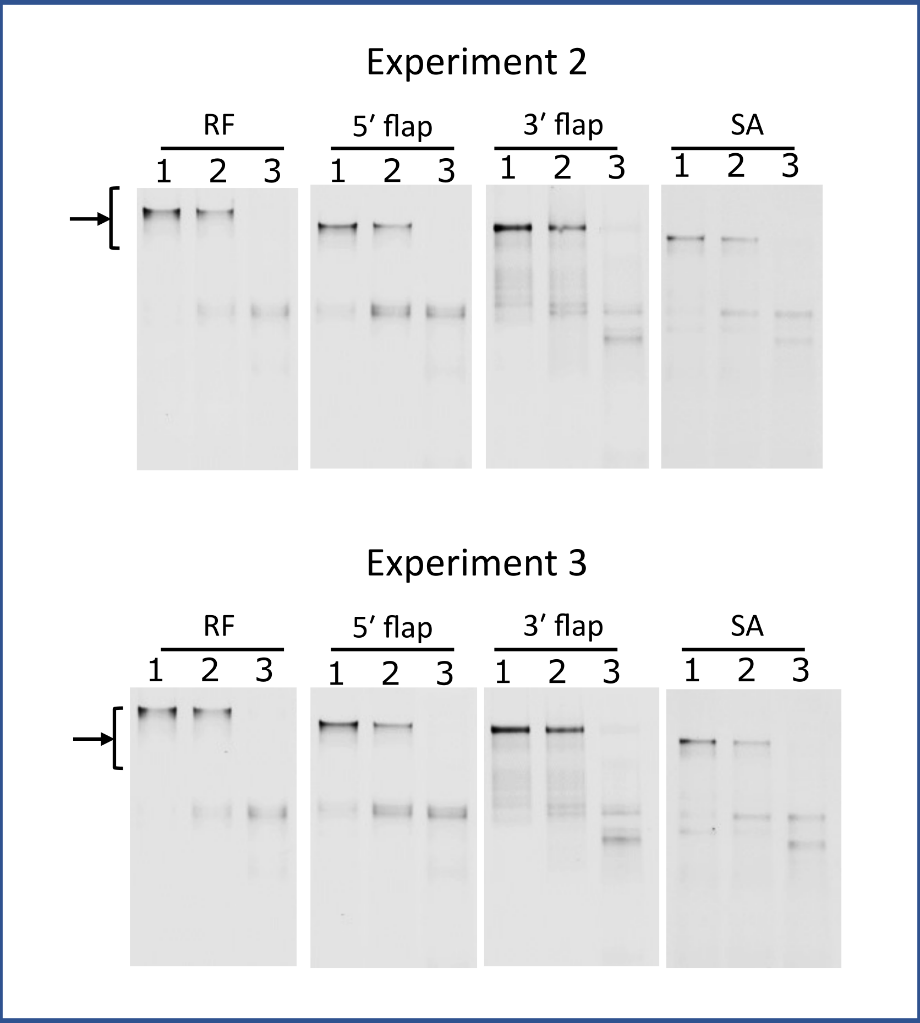


**SI Figure 10. Substrate specificity of *At*-HIGLE.** Rest of the activity assays on synthetic DNA substrates: replication fork (RF), 5′ flap, 3′ flap, and splayed arm (SA) used for quantitation of product formation reported in Figure 3.


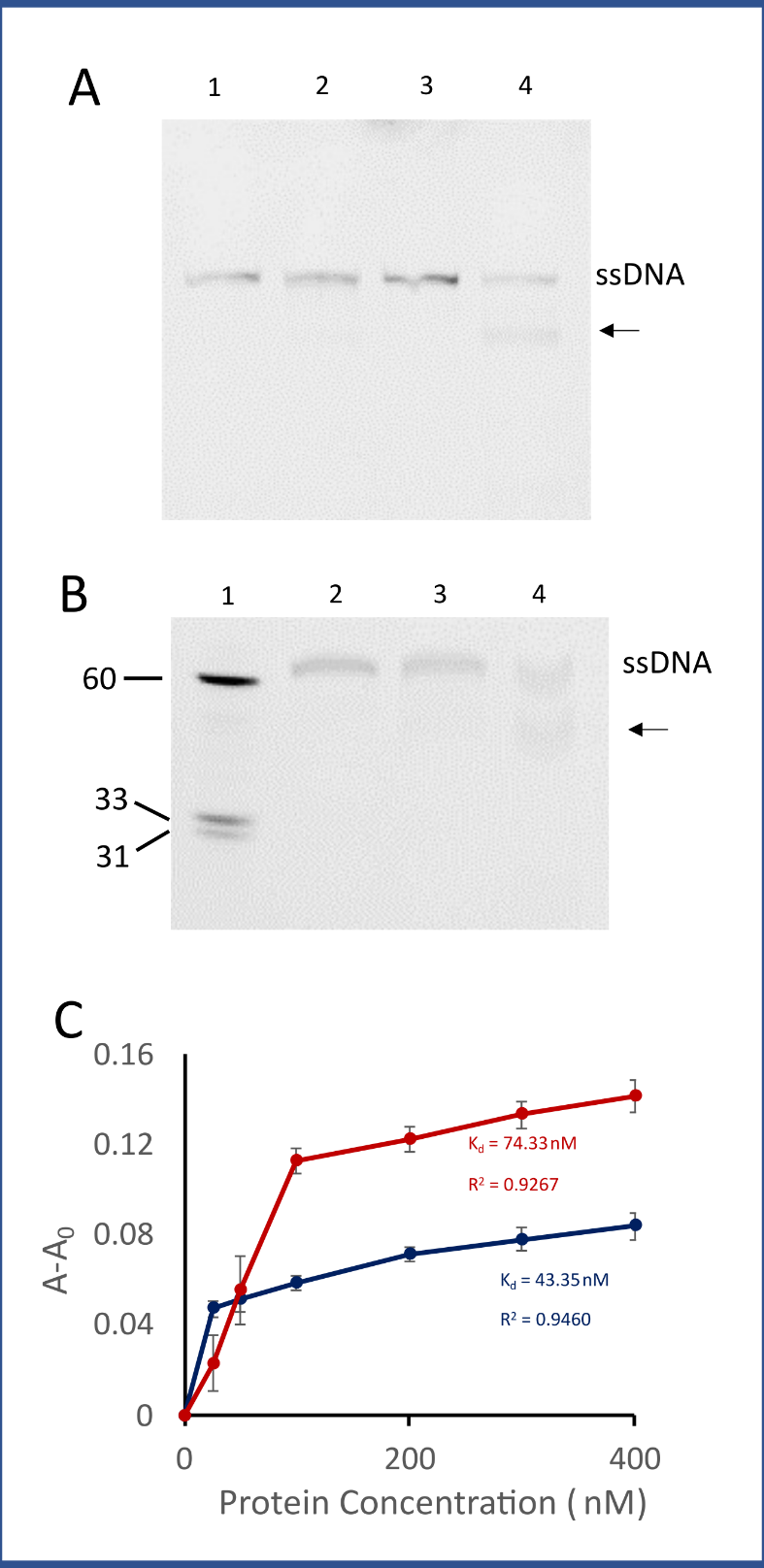


**SI Figure 11. Catalytic activity and binding on single-stranded DNA.** **(A)** A 60-mer single stranded DNA labeled with 6-FAM at 5′ end was subjected to catalytic activity at 37 ⁰C by *At*-HIGLE (lane 1: 0min; lane 2: 90 min) and *At*-HIGLE^1-183^ (lane 3: 0min; lane 4: 90 min) and the products were resolved on a 10 % TBE-native PAGE and scanned for 6-FAM signal. The product is shown with an arrow. **(B)** The 90 min reaction was run on a 12% TBE-Urea PAGE and scanned for 6-FAM signal. Lane 1: markers, lane 2: substrate alone, lane 3: 90 min reaction with *At-*HIGLE, lane 4: 90 min reaction with *At*-HIGLE^1-183^. The product is shown with an arrow. **(C)** Binding studies with single-stranded DNA in the presence of *At*-HIGLE (full length) (blue) and *At*-HIGLE^1-183^ (red) using fluorescence anisotropy with standard error bars. Signal for 6-FAM was used for anisotropy experiments. Y-axis is shown as change in anisotropy (A-A_0_), where A is observed anisotropy and A_0_ is anisotropy of DNA substrate alone. All experiments were done in triplicates.


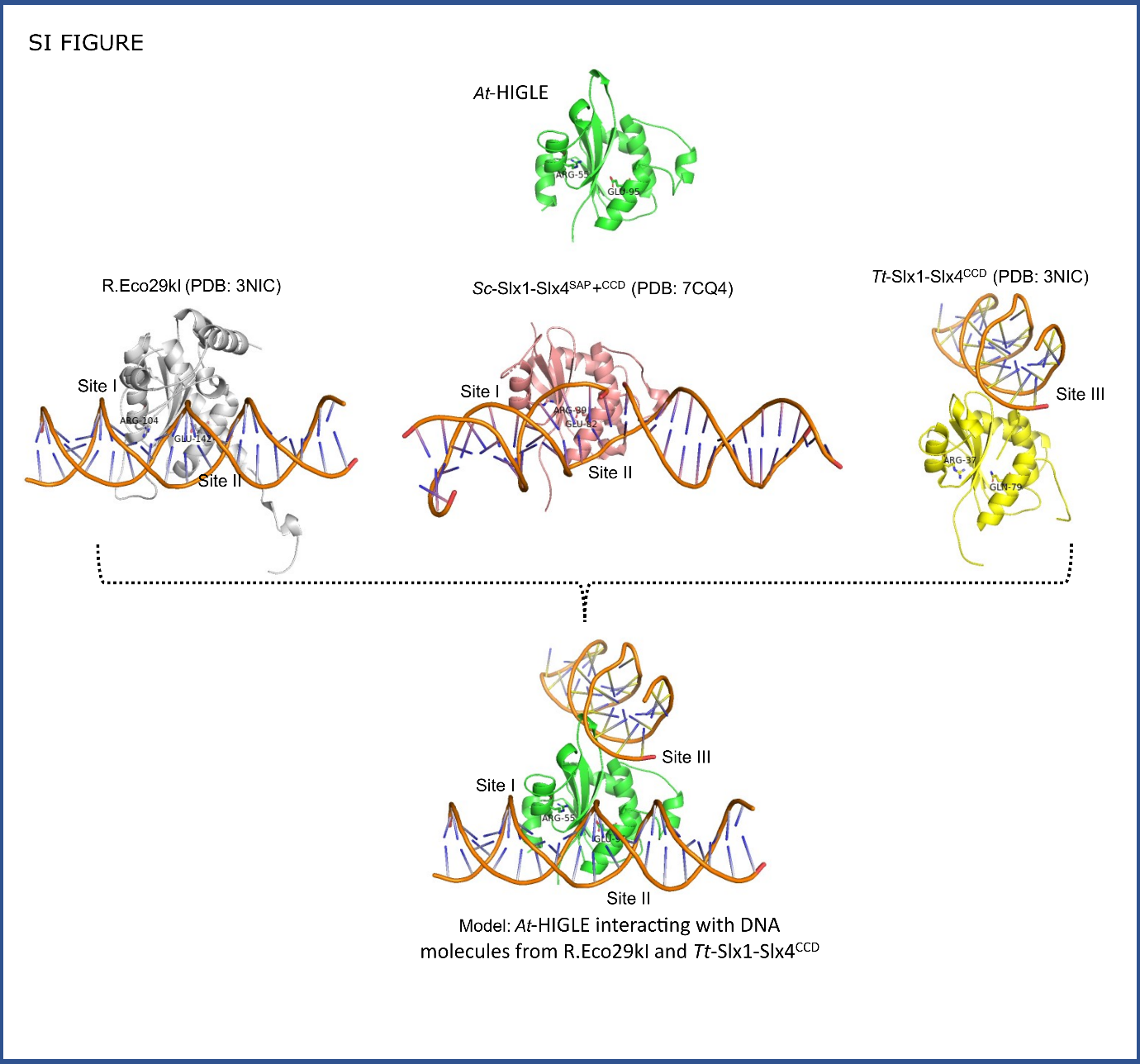


**SI Figure 12. *At*-HIGLE-DNA substrate model.** Nuclease domains of R.Eco29kI (PDB: 3NIC), *Sc*-Slx1-Slx4^SAP+CCD^ (PDB: 7CQ4), and *Tt*-Slx1-SLX4^CCD^ (PDB: 6SEI) along with bound DNA fragments were superimposed on the nuclease domain of *At*-HIGLE based on conservation of the active site. DNA fragments bound to R.Eco29kI and *Tt*-Slx1-Slx4^CCD^ were then retained over the *At*-HIGLE structure. DNA molecules bound to R.Eco29kI (PDB: 3NIC), *Sc*-Slx1-Slx4^SAP+CCD^ (PDB: 7CQ4), and *Tt*-Slx1-SLX4^CCD^ (PDB: 6SEI) provided clues about *At*-HIGLE residues involved in branched DNA substrate interaction. The DNA binding residues are organized in three distinct sites: site I, site II, and site III.


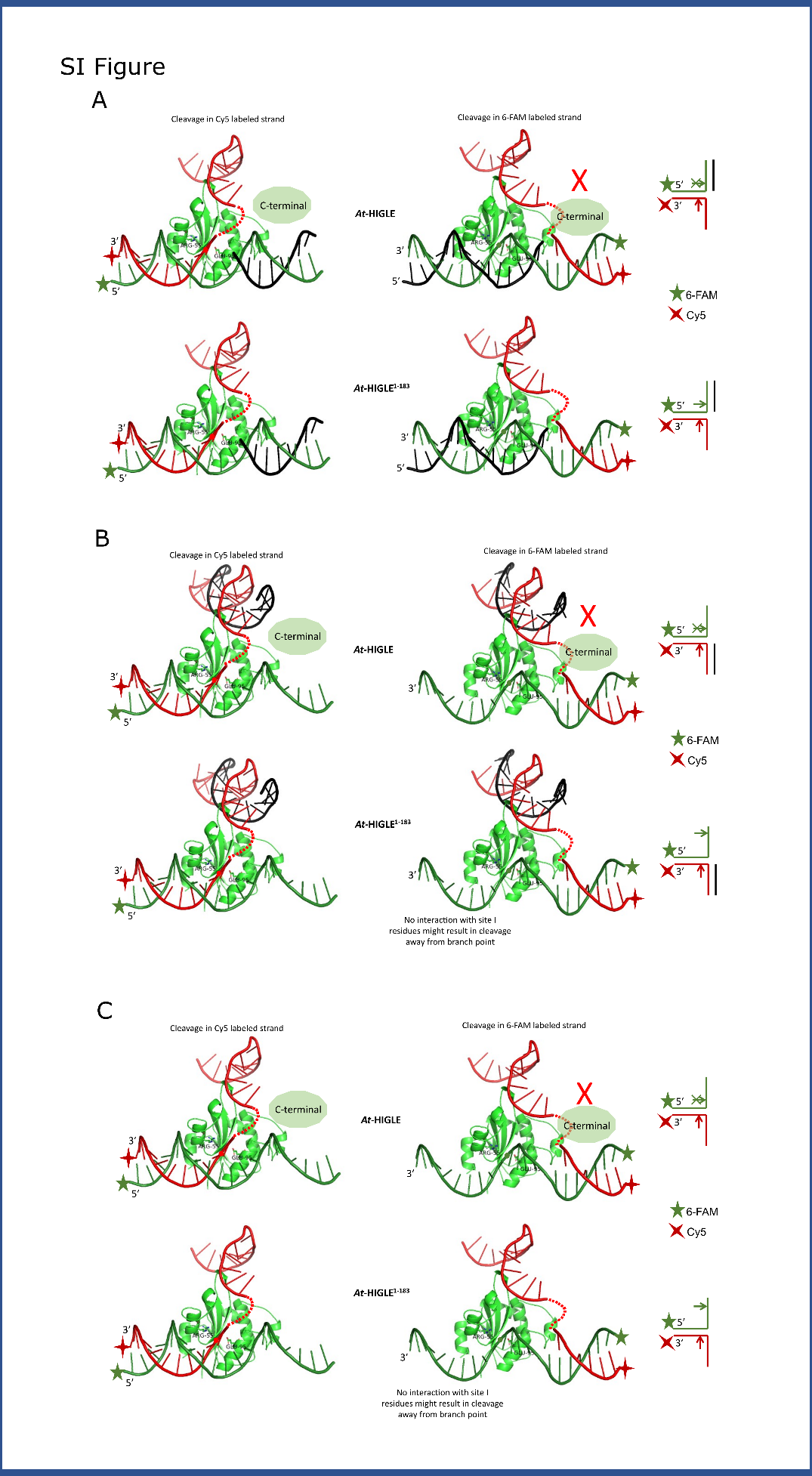


**SI Figure 13.** Models explaining cleavage of 6-FAM and Cy5 labeled strands of various branched DNA substrates. **(A)** Cleavage of 5′ flap. **(B)** Cleavage of 3′ flap. **(C)** Cleavage of splayed arm substrate. The scissile phosphate in 6-FAM labeled and Cy5 labeled DNA strands are shown with green and red circles respectively.


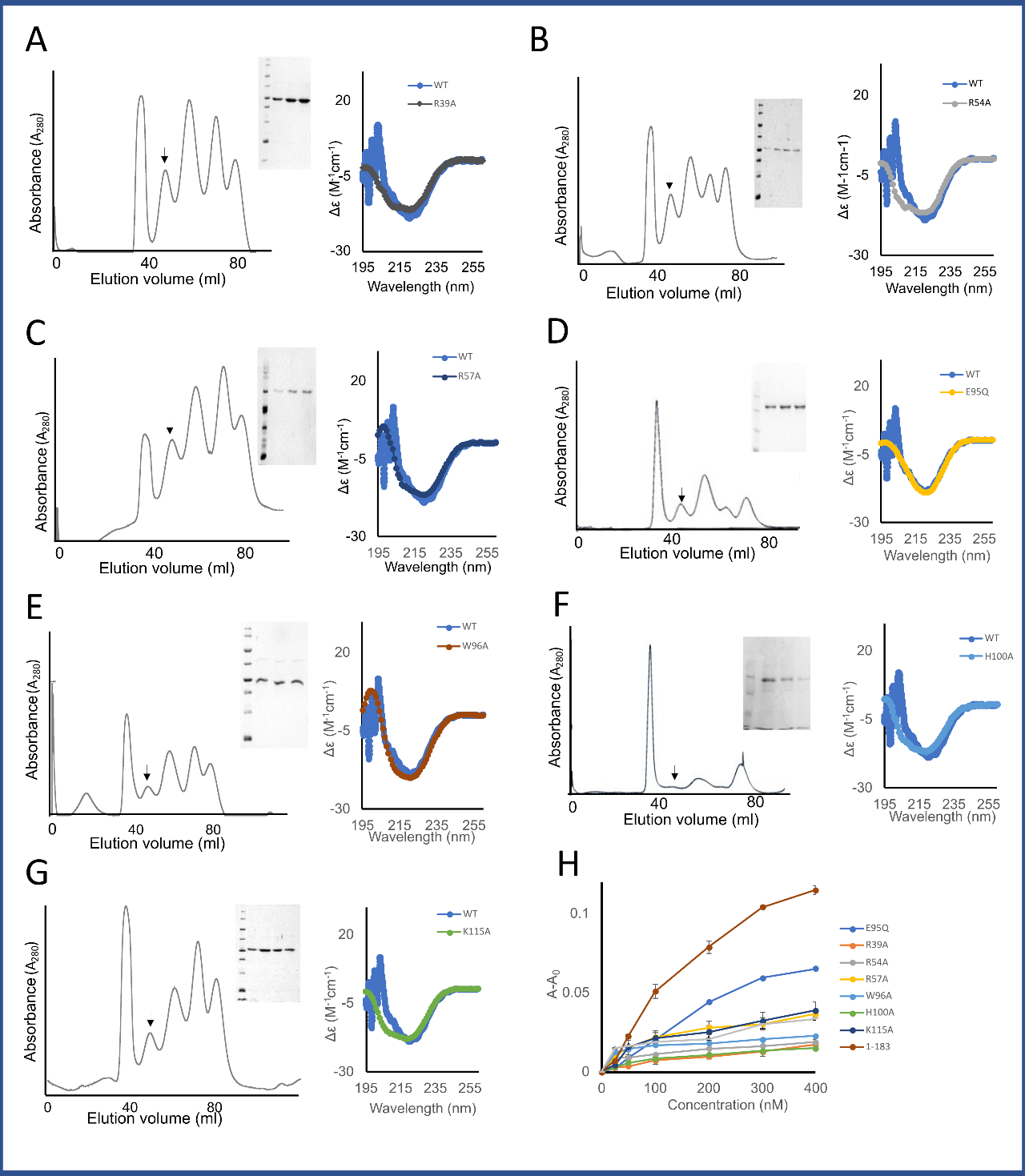


**SI Figure 14. Characterization of *At*-HIGLE mutants used in the study.** **(A-G)** The last protein purification step for each mutant involved size exclusion chromatography (Sephacryl 200). Gel filtration profile and quality of purified protein on an SDS-PAGE have been shown. The SDS-PAGE profiles of rest of the peaks in Size exclusion chromatogram are same as *At*-HIGLE (WT) as shown in SI Figure 15. The arrow marks the peak of elution of the protein. Each panel has a circular dichroism spectrum of the mutant compared with circular dichroism of Wild type *At*-HIGLE (WT). A: *At*-HIGLE^R39A^; B: *At*-HIGLE^R54A^; C: *At*-HIGLE^R57A^; D: *At*-HIGLE^E95Q^; E: *At*-HIGLE^W96A^; F: *At*-HIGLE^H100A^; G: *At*-HIGLE^K115A^. **(H)** The HJ binding potential of various proteins used in the study using fluorescence anisotropy with standard error bars. Signal for 6-FAM was used for anisotropy experiments. Y-axis is shown as change in anisotropy (A-A_0_), where A is observed anisotropy and A_0_ is anisotropy of DNA substrate alone. The data for *At*-HIGLE^E95Q^ and *At*-HIGLE^1-183^ is also presented in Figure 2. Protein concentration is plotted on X-axis. All experiments were done in triplicates.


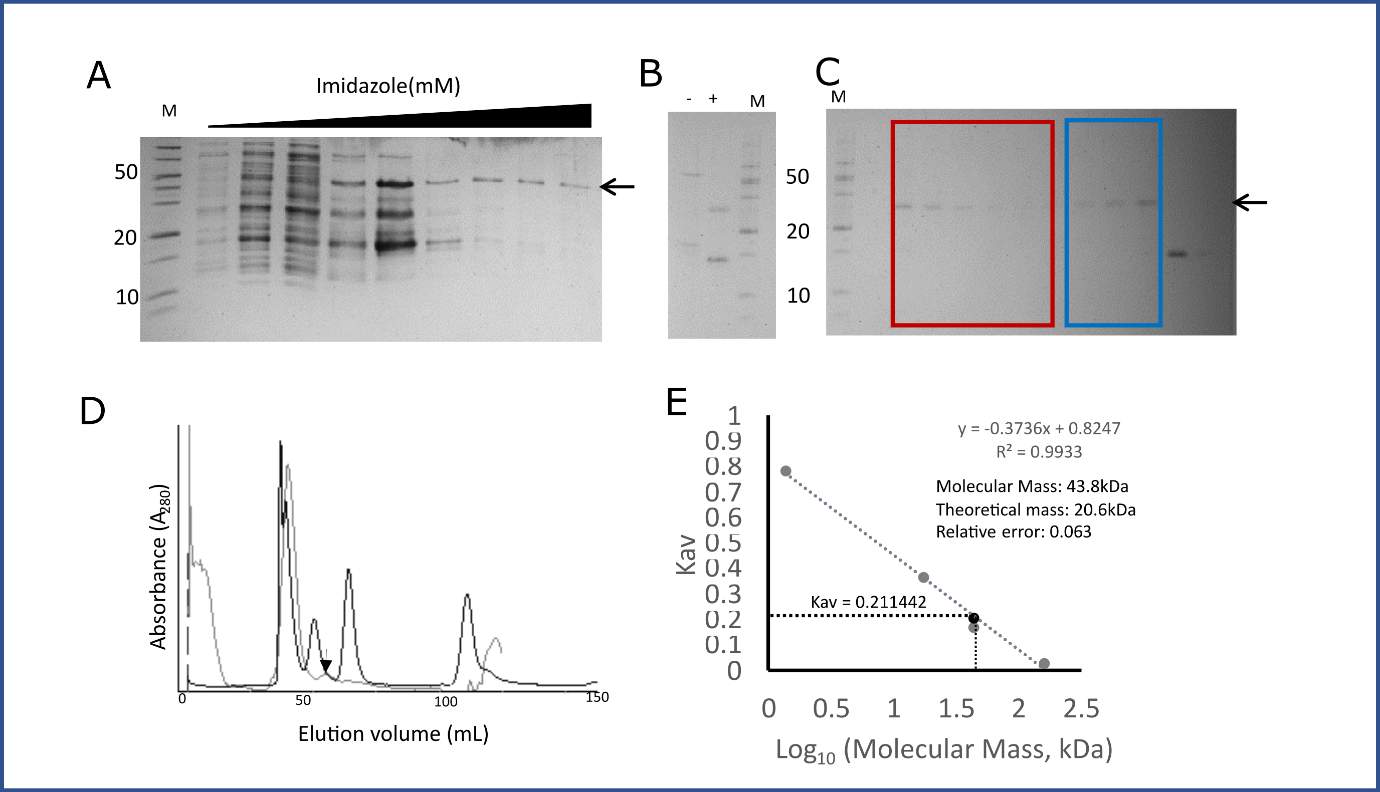


**SI Figure 15. Purification of C-terminal domain of *At*-HIGLE^184-368^.** **(A)** SDS-PAGE profile showing purification on Ni-NTA column. Arrow depicts the migration of His-tagged protein **(B)** SDS-PAGE profile showing removal of His-tag using SUMO protease. – and + represent samples before and after His-tag removal, respectively. **(C)** SDS-PAGE profile showing various fractions after purification using size exclusion chromatography on a Sephacryl 100 column. Red and blue boxes represent samples corresponding to void volume peak and soluble fractions, respectively. Arrow depicts the migration of protein without His-tag. **(D)** Size-exclusion chromatogram of *At*-HIGLE^184-368^ in grey superimposed with gel filtration markers (in black). **(E)** Estimation of the oligomeric weight of *At*-HIGLE^184-368^ from a standard curve generated using gel filtration markers (Vitamin B12, Myoglobin, Ovalbumin, and gamma globulin). Kav was calculated as (Ve-Vo)/(Vt-Vo), where Ve, Vo, and Vt are elution volume, void volume, and total volume, respectively. Thyroglobulin (670 kDa) was used to determine the column's void volume.


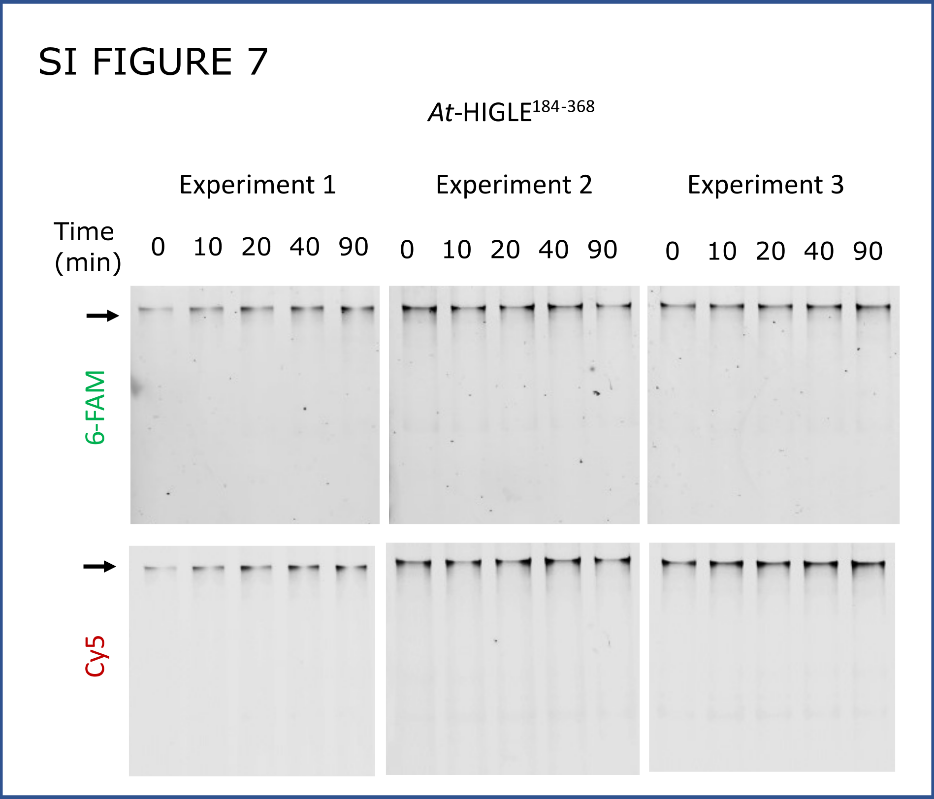


**SI Figure 16. Catalytic activity of *At*-HIGLE^184-368^ on Holliday Junction.** Catalytic activity of C-terminal region of *At*-HIGLE (*At*-HIGLE^184-368^) on synthetic Holliday Junction. The Substrate is indicated by arrows.


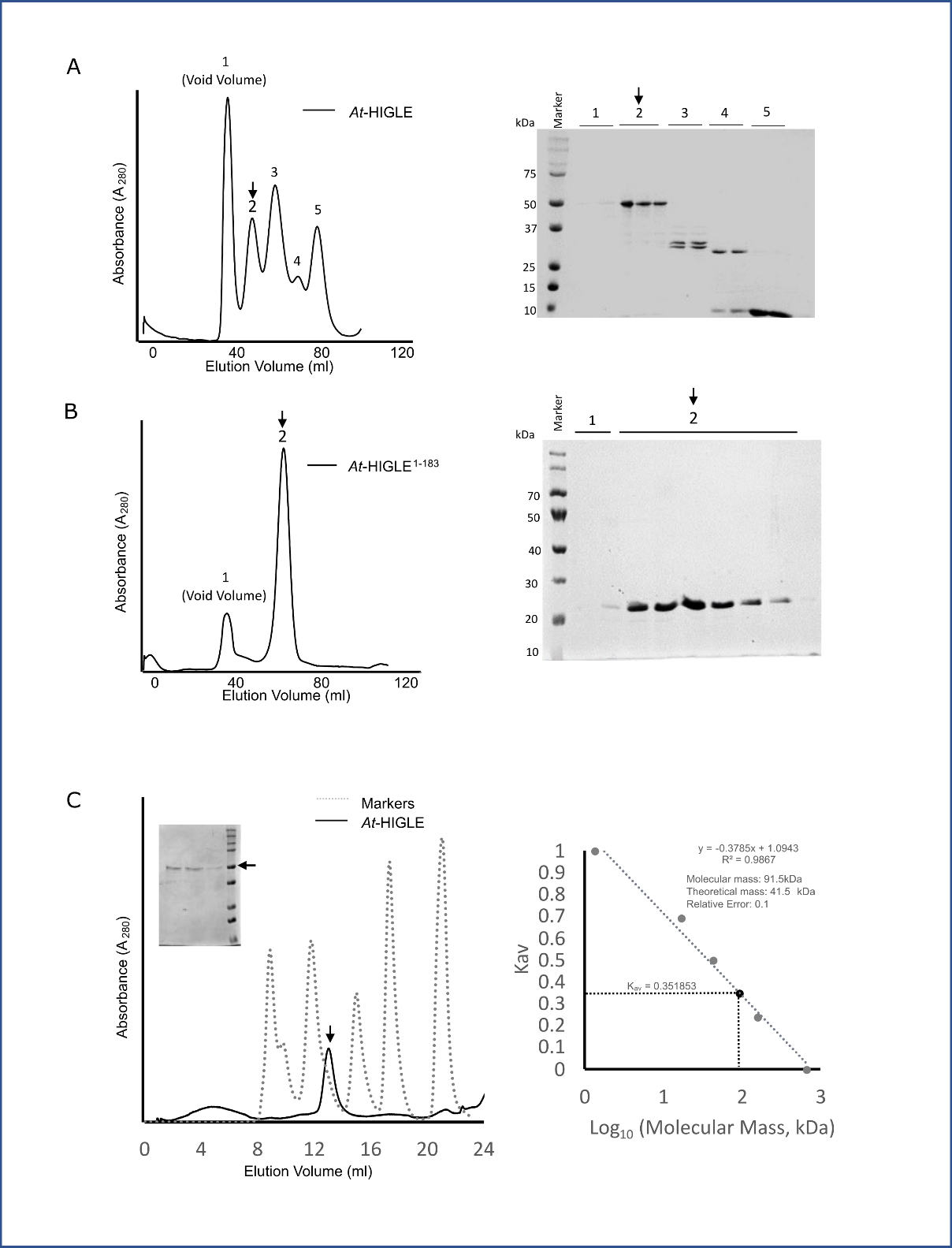


**SI Figure 17. Size exclusion chromatogram and corresponding SDS-PAGE profiles. (A)** Size-exclusion chromatogram of *At*-HIGLE purified on a Sephacryl S200 gel filtration along with corresponding 12% SDS-PAGE profile. The various peaks are marked with numbers. The peak corresponding to *At*-HIGLE is demarcated by an arrow. The chromatogram reported here is same as reported in Figure 5. **(B)** Size-exclusion chromatogram of *At*-HIGLE^1-183^ purified on a Sephacryl S100 gel filtration along with corresponding 12% SDS-PAGE profile. The various peaks are marked with numbers. The peak corresponding to *At*-HIGLE^1-183^ is demarcated by an arrow. The chromatogram reported here is same as reported in Figure 5. **(C)** Size-exclusion chromatogram (black) of purified *At*-HIGLE re-run on a Superdex 200 increase 10/300 column superimposed with gel filtration markers (broken grey). The peak corresponding to *At*-HIGLE is demarcated by an arrow. The fractions from the demarcated peak were run on a 12% SDS-PAGE. Estimation of the oligomeric weight of *At*-HIGLE from a standard curve generated using gel filtration markers (Vitamin B12, Myoglobin, Ovalbumin, and gamma globulin. Kav was calculated as (Ve-Vo)/(Vt-Vo) where Ve, Vo, and Vt are elution volume, void volume, and total volume, respectively. Thyroglobulin (670 kDa) was used to determine the column's void volume.


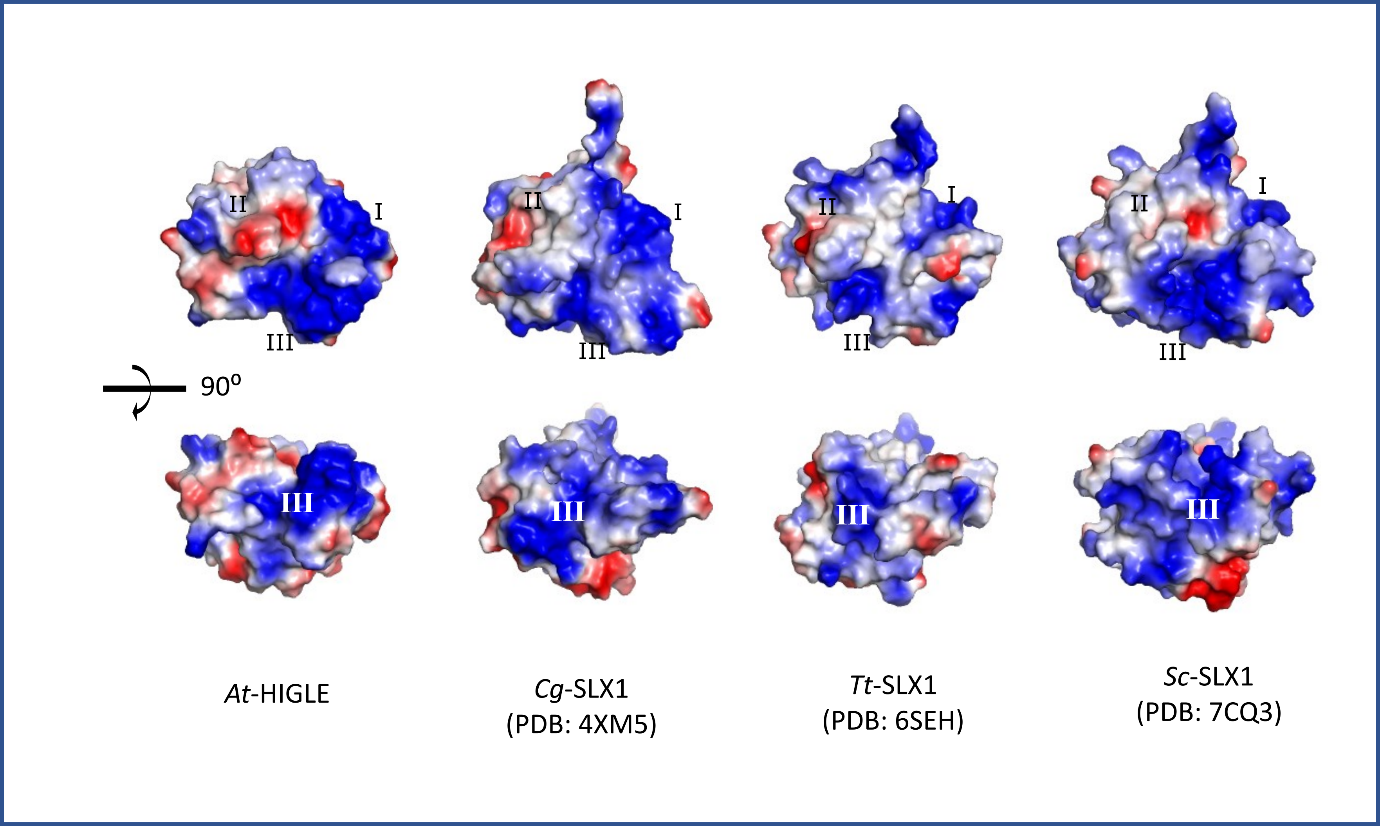


**SI Figure 18. Surface charge distribution on the nuclease domains *At*-HIGLE and fungal SLX1 proteins.** *At*-HIGLE and SLX1 proteins have surface-exposed positively charged residues organized into distinct patches for interaction with various arms of branched DNA substrates. The three sites of *At*-HIGLE predicted to interact with various arms of branched DNA substrates are labeled as I, II, and III. The corresponding sites on *Cg*-SLX1, *Tt*-SLX1, and *Sc*-SLX1 are also demarcated.

**SI Table 1.** **Oligonucleotides used for Site-Directed Mutagenesis**

| Name | Sequence |
| --- | --- |
| *At*-HIGLE-R54A-R | GCTGACGAATGCGAGCGCGCGGATTCACGG |
| *At*-HIGLE-R54A-F | CCGTGAATCCGCGCGCTCGCATTCGTCAGC |
| *At*-HIGLE-R57A-R | CCATTATGCTGAGCAATGCGACGGCGCGGATTCAC |
| *At*-HIGLE-R57A-F | GTGAATCCGCGCCGTCGCATTGCTCAGCATAATGG |
| *At*-HIGLE-W96A-R | GCTGCCAGGCCGCTTCAAACTGCAGTGCACTCAC |
| *At*-HIGLE-W96A-F | GTGAGTGCACTGCAGTTTGAAGCGGCCTGGCAGC |
| *At*-HIGLE-H100A-R | CACTTTCGCGCGGAGCCTGCCAGGCCCATT |
| *At*-HIGLE-H100A-F | AATGGGCCTGGCAGGCTCCGCGCGAAAGTG |
| *At*-HIGLE-R39A-R | GTCTGGCCTTTATGGGCCGGACTCAGACTGGT |
| *At*-HIGLE-R39A-F | ACCAGTCTGAGTCCGGCCCATAAAGGCCAGAC |
| *At*-HIGLE-K115A-R | GGCAACACCACTAAAACTTGCAAAGGCTGCGGCTGCTTCG |
| *At*-HIGLE-K115A-F | CGAAGCAGCCGCAGCCTTTGCAAGTTTTAGTGGTGTTGCC |
| *At*-HIGLE-E95Q-F | CAGGCCCATTCAAACTCCAGTGCACTCACATTG |
| *At*-HIGLE-E95Q-R | CAATGTGAGTGCACTGGAGTTTGAATGGGCCTG |
| *At*-HIGLE-1-183-F | CTTTCTTCATCTTCCGGCTGCTAACTATCATCAACTTTGGTAAAATACTGCAGATCT |
| *At*-HIGLE-1-183-R | AGATCTGCAGTATTTTACCAAAGTTGATGATAGTTAGCAGCCGGAAGATGAAGAAAG |
| *At*-HIGLE-184-368-F | ACAAACCGGTGGATCCAGTCAGCCGGAAGATG |
| *At*-HIGLE-184-368-R | CATCTTCCGGCTGACTGGATCCACCGGTTTGT |

**SI Table 2. Details of DNA oligonucleotides and substrates used in nuclease assays.**

**SI Table 2A.** Sequences of DNA oligonucleotides used in annealing synthetic substrates for activity tests

| **Name** | **Sequence (5′ to 3′)** |
| --- | --- |
| X0-1 | ACGCTGCCGAATTCTACCAGTGCCTTGCTAGGACATCTTTGCCCACCTGCAGGTTCACCC |
| X0-2 | GGGTGAACCTGCAGGTGGGCAAAGATGTCCATCTGTTGTAATCGTCAAGCTTTATGCCGT |
| X0-3 | ACGGCATAAAGCTTGACGATTACAACAGATCATGGAGCTGTCTAGAGGATCCGACTATCG |
| X0-4 | CGATAGTCGGATCCTCTAGACAGCTCCATGTAGCAAGGCACTGGTAGAATTCGGCAGCGT |
| X0-2.30 | GGGTGAACCTGCAGGTGGGCAAAGATGTCC |
| X0-3.30 | CATGGAGCTGTCTAGAGGATCCGACTATCG |
| X0-1^f^ | [6-FAM] ACGCTGCCGAATTCTACCAGTGCCTTGCTAGGACATCTTTGCCCACCTGCAGGTTCACCC |
| X0-4^c^ | CGATAGTCGGATCCTCTAGACAGCTCCATGTAGCAAGGCACTGGTAGAATTCGGCAGCGT (Cy5) |

**SI Table 2B.** Combination of DNA oligonucleotides used to generate various joint DNA molecules

| **Name** | **Unlabeled substrate** | **Labeled substrate** |
| --- | --- | --- |
| Holliday junction (HJ) | X0-1, X0-2, X0-3, X0-4 | X0-1^f^, X0-2, X0-3, X0-4^c^ |
| 5′ flap | X0-1, X0-2.30, X0-4 | X0-1 ^f^, X0-2.30, X0-4^c^ |
| 3′ flap | X0-1, X0-3.30, X0-4 | X0-1 ^f^, X0-3.30, X0-4^c^ |
| Replication fork (RF) | X0-1, X0-2.30, X0-3.30, X0-4 | X0-1 ^f^, X0-2.30, X0-3.30, X0-4^c^ |
| Splayed arm (SA) | X0-1, X0-4 | X0-1 ^f^, X0-4^c^ |

**SI Table 2C.** Sequences of DNA oligonucleotides used for generating DNA ladders.

| **Oligonucleotides used for generating ladder** | |
| --- | --- |
| X0-1^f^ | [6-FAM] ACGCTGCCGAATTCTACCAGTGCCTTGCTAGGACATCTTTGCCCACCTGCAGGTTCACCC |
| X0-1^f^_31 | [6-FAM] ACGCTGCCGAATTCTACCAGTGCCTTGCTAG |
| X0-1^f^_33 | [6-FAM] ACGCTGCCGAATTCTACCAGTGCCTTGCTAGGA |
| X0-4^c^ | CGATAGTCGGATCCTCTAGACAGCTCCATGTAGCAAGGCACTGGTAGAATTCGGCAGCGT (Cy5) |
| X0-4^c^_27 | CAAGGCACTGGTAGAATTCGGCAGCGT (Cy5) |
| X0-4^c^_29 | AGCAAGGCACTGGTAGAATTCGGCAGCGT (Cy5) |
| X0-4^c^_31 | GTAGCAAGGCACTGGTAGAATTCGGCAGCGT (Cy5) |

**Schematic representation of substrates used in nuclease assays***

HJ

X0-1

X0-2

X0-4

X0-3

RF

X0-1

X0-2.30

X0-4

X0-3.30

3ʹ Flap

X0-1

X0-4

X0-3.30

5ʹ Flap

X0-1

X0-2.30

X0-4

SA

X0-1

X0-4

*****Green represents 6-FAM and red represents Cy5 labeled strands

**SI Table 3. Details of DNA oligonucleotides and substrates used in fluorescence anisotropy.**

**SI Table 3A.** Sequences of DNA oligonucleotides used in annealing synthetic substrates for fluorescence anisotropy

| **Name** | **Sequence (5′ to 3′)** |
| --- | --- |
| F0-1 | ACCAGTGCCTTGCTAGGACATCTTTGCCCA |
| F0-2 | TGGGCAAAGATGTCCATCTGTTGTAATCGT |
| F0-3 | ACGATTACAACAGATCATGGAGCTGTCTAG |
| F0-4 | CTAGACAGCTCCATGTAGCAAGGCACTGGT |
| F0-2.30 | TGGGCAAAGATGTCC |
| F0-3.30 | CATGGAGCTGTCTAG |
| F0-1^f^ | [6-FAM] ACCAGTGCCTTGCTAGGACATCTTTGCCCA |

**SI Table 3B.** Combination of DNA oligonucleotides used to generate various joint DNA molecules

| **Name** | **Unlabeled** | **Labeled** |
| --- | --- | --- |
| Holliday junction | F0-1, F0-2, F0-3, F0-4 | F0-1^f^, F0-2, F0-3, F0-4 |
| 5′ flap | F0-1, F0-2.30, F0-4 | F0-1^f^, F0-2.30, F0-4 |
| 3′ flap | F0-1, F0-3.30, F0-4 | F0-1^f^, F0-3.30, F0-4 |
| Replication fork | F0-1, F0-2.30, F0-3.30, F0-4 | F0-1^f^, F0-2.30, F0-3.30, F0-4 |
| Splayed arm | F0-1, F0-4 | F0-1^f^, F0-4 |

Schematic representation of substrates for fluorescence anisotropy*

HJ

F0-1

F0-2

F0-4

F0-3

RF

F0-1

F0-2.30

F0-4

F0-3.30

3ʹ flap

F0-1

F0-4

F0-3.30

5ʹ flap

F0-1

F0-2.30

F0-4

SA

F0-1

F0-4

*****Green represents 6-FAM labeled strand.

**REFERENCES**

1. Thompson, J.D., Higgins, D.G. and Gibson, T.J. (1994) CLUSTAL W: improving the sensitivity of progressive multiple sequence alignment through sequence weighting, position-specific gap penalties and weight matrix choice. *Nucleic Acids Res*, **22**, 4673-4680.

2. Jones, D.T. and Cozzetto, D. (2015) DISOPRED3: precise disordered region predictions with annotated protein-binding activity. *Bioinformatics*, **31**, 857-863.

3. Jones, D.T. (1999) Protein secondary structure prediction based on position-specific scoring matrices. *J Mol Biol*, **292**, 195-202.

4. Buchan, D.W.A. and Jones, D.T. (2019) The PSIPRED Protein Analysis Workbench: 20 years on. *Nucleic Acids Res*, **47**, W402-W407.
